# Supplementary material for: Homeostatic regulation of STING by retrograde membrane traffic to the ER
Source: Nat Commun. 2021 Jan 4;12:61. doi: 10.1038/s41467-020-20234-9 (PMC7782846; doi:10.1038/s41467-020-20234-9)
Supplement: Supplementary file 1 — Supplementary Information [file 41467_2020_20234_MOESM1_ESM.pdf]

# Supplementary Figure 1

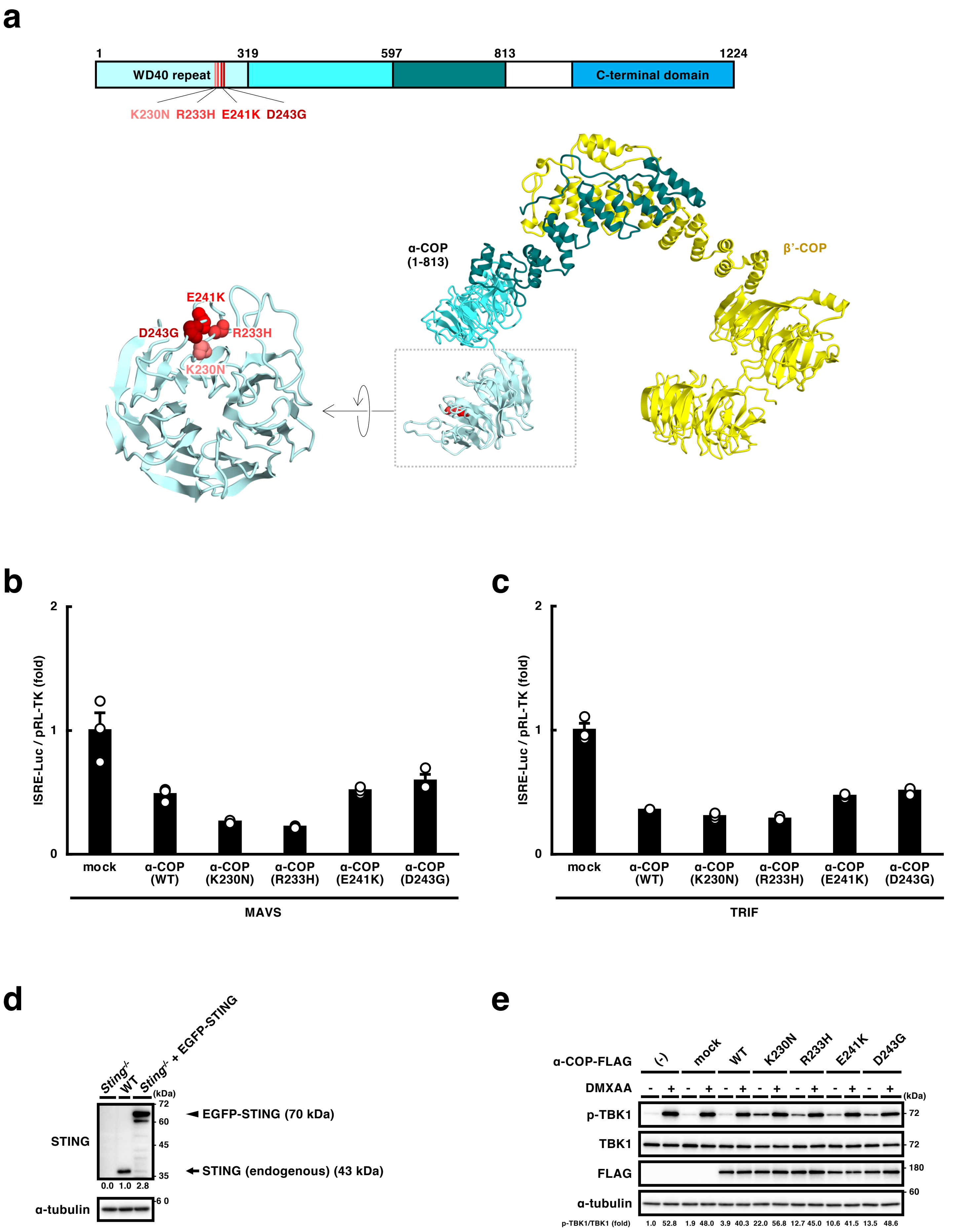


**Supplementary Figure. 1 | Supplementary materials related to Fig. 1.**

**a**, A schematic of α-COP protein and its domains are shown. The amino acid positions corresponding to the mutations identified in COPA syndrome are indicated. Three-dimensional structure is from PDB ID 5NZR. Light cyan: α-COP (WD40 repeat, aa 1-319), cyan: α-COP (aa 320-597), dark cyan: α-COP (aa 598-813), yellow: β'-COP. **b**, **c**, HEK293T cells were transfected as indicated, together with the ISRE (also known as PRDIII or IRF-E)-luciferase reporter. Luciferase activity was then measured. Data represent mean±s.e.m. of three independent experiments. **d**, EGFP-STING were stably expressed in *Sting*$^{-/-}$ MEFs. The expression levels of STING were analyzed by western blot. **e**, The α-COP variants expressing MEFs were stimulated with DMXAA (25 μg/mL) for 1 h. Cell lysates were prepared and analyzed by western blot. The results suggested that the STING signalling in cells expressing the disease-causative α-COP can be further activated two- to three-fold with the STING ligand.

# Supplementary Figure 2

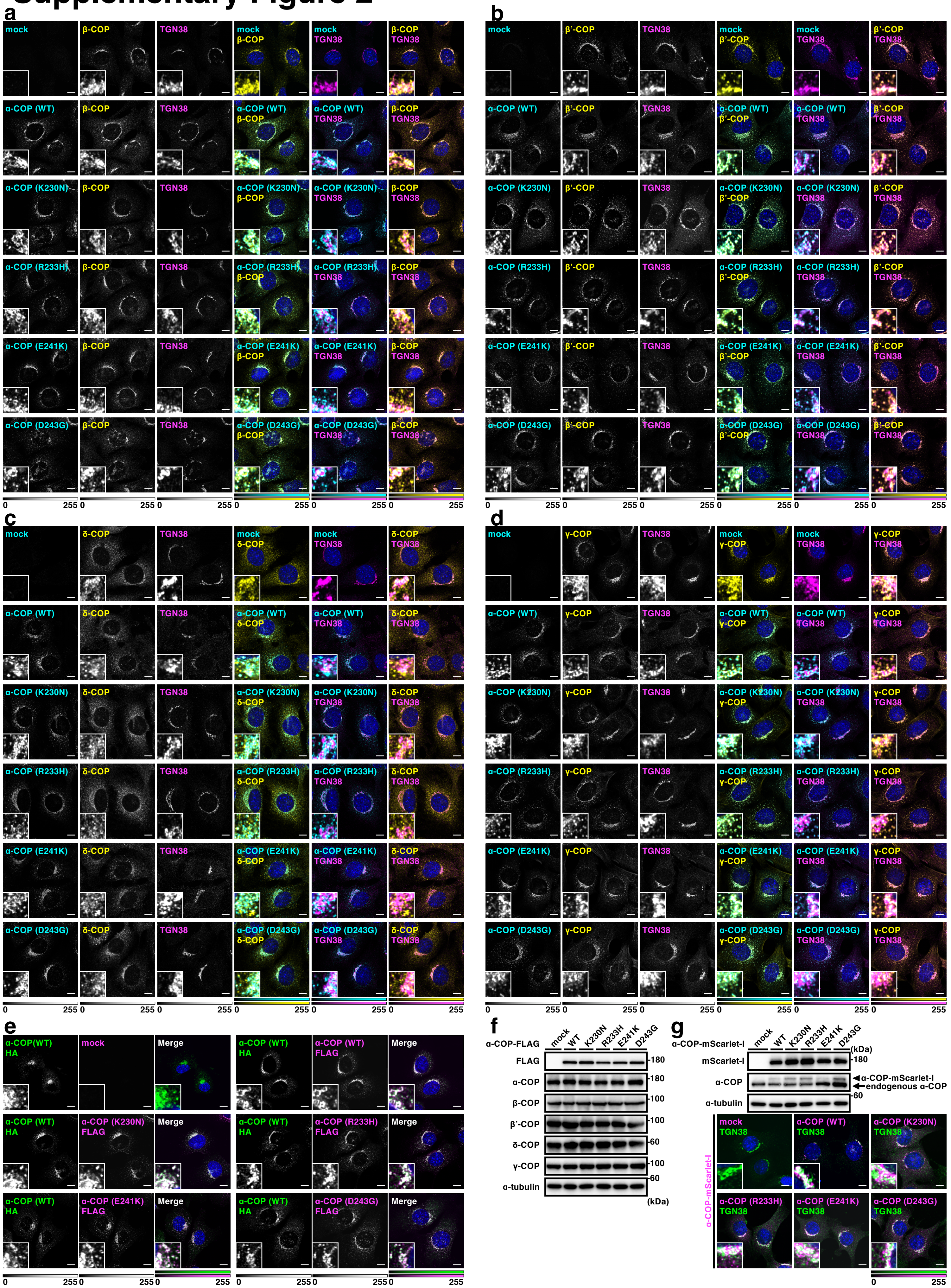


**Supplementary Figure 2 | Expression of the α-COP variants do not affect the localization of endogenous COP-I subunits.**
**a**-**d**, α-COP-FLAG were stably expressed in MEFs. Cells were fixed, permeabilized, and stained for β-COP (**a**), β’-COP (**b**), δ-COP (**c**), or γ-COP (**d**). α-COP-FLAG (cyan), TGN38 (a Golgi protein; magenta) and nuclei (blue) were also stained. The magnified image of the perinuclear region is shown in the lower left box. Scale bars, 10 µm. **e**, α-COP-FLAG (WT or mutant) and α-COP-HA (WT) were stably expressed in MEFs. Cells were fixed, permeabilized, and stained with HA and FLAG antibody. The magnified image of the perinuclear region is shown in the lower left box. Scale bars, 10 µm. **f**, The expression levels of several COP-I subunits in MEFs expressing the α-COP variants were analyzed by western blot. **g**, α-COP-mScarlet-I were stably expressed in MEFs. The expression levels of endogenous or exogenous α-COP were analyzed by western blot (upper panel). All the α-COP-mScarlet-I were co-localized with TGN38 (lower panel). Scale bars, 10 µm.

# Supplementary Figure 3

**a**

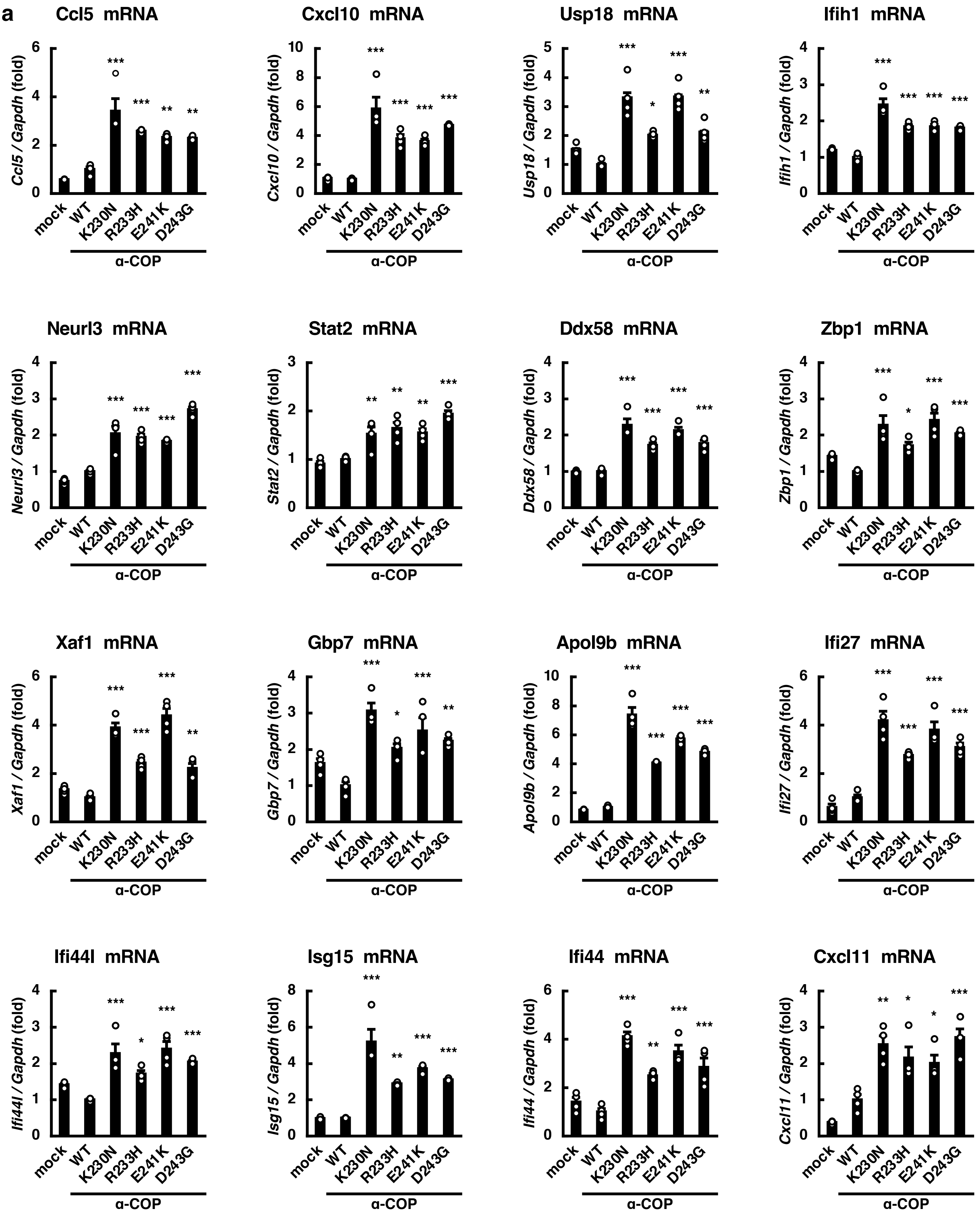


**b**

| | *Ccl5* | *Cxcl10* | *Isg15* | *Ifi44* | *Usp18* | *Ifih1* | *Neurl3* | *Stat2* | *Ddx58* | *Zbp1* | *Cxcl11* | *Xaf1* | *Gbp7* | *Apol9b* | *Ifi27* | *Ifi44l* |
|---|---|---|---|---|---|---|---|---|---|---|---|---|---|---|---|---|
| WT-K230N | < 0.0001 | < 0.0001 | < 0.0001 | < 0.0001 | < 0.0001 | < 0.0001 | < 0.0001 | 0.0098 | < 0.0001 | 0.0001 | 0.0012 | < 0.0001 | < 0.0001 | < 0.0001 | < 0.0001 | 0.0001 |
| WT-R233H | 0.0009 | 0.0003 | 0.0022 | 0.0023 | 0.0174 | < 0.0001 | < 0.0001 | 0.0014 | 0.0004 | 0.0231 | 0.0158 | 0.0003 | 0.0138 | < 0.0001 | 0.0006 | 0.0231 |
| WT-E241K | 0.0051 | 0.0007 | < 0.0001 | < 0.0001 | < 0.0001 | < 0.0001 | 0.0001 | 0.0058 | < 0.0001 | < 0.0001 | 0.0422 | < 0.0001 | 0.0003 | < 0.0001 | < 0.0001 | < 0.0001 |
| WT-D243G | 0.0071 | < 0.0001 | 0.0007 | 0.0002 | 0.0073 | < 0.0001 | < 0.0001 | < 0.0001 | 0.0002 | 0.0007 | 0.0003 | 0.0017 | 0.0031 | < 0.0001 | 0.0001 | 0.0007 |

**Supplementary Figure 3 | The expressions of innate immune genes are increased in the α-COP variants-expressing cells.**
**a**, Quantitative real-time PCR (qRT-PCR) of the expression of innate immune genes in the α-COP variant expressing MEFs. Indicated gene expression was normalized on the basis of GAPDH content and fold change compared to α-COP (WT) was plotted. Data are mean±s.e.m. from four independent experiments. Statistical significances between α-COP (WT) and the α-COP variants were determined with one-way analysis of variance followed by Tukey–Kramer *post hoc* test. *$P$<0.05; **$P$<0.01; ***$P$<0.001. **b**, Exact $P$ values in **a** are indicated.

# Supplementary Figure 4

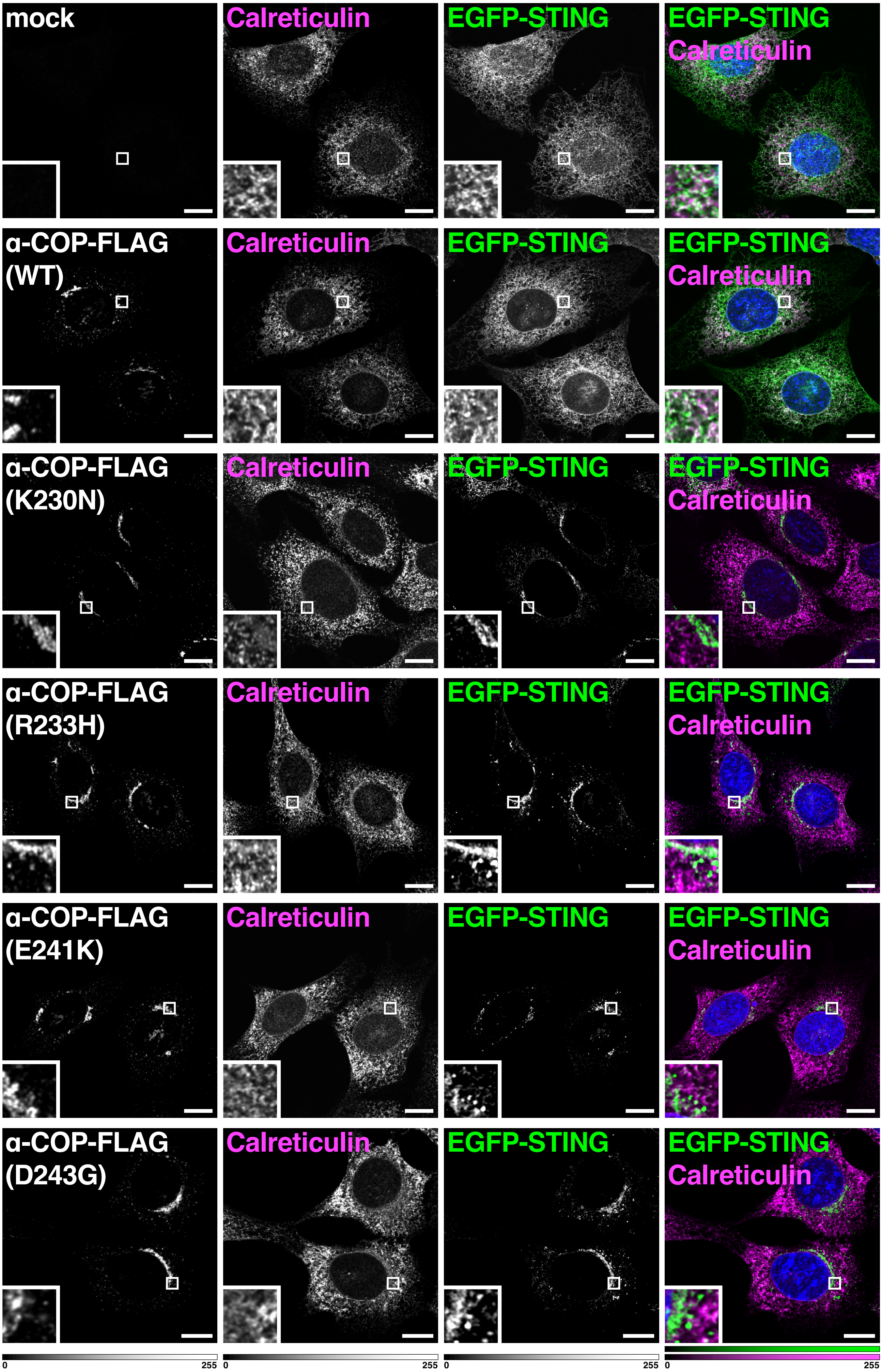


**Supplementary Figure 4 | STING translocates from the ER in the α-COP variants-expressing cells.**
α-COP-FLAG and EGFP-STING were stably expressed in *Sting*$^{-/-}$ MEFs. Cells were fixed, permeabilized, and stained for calreticulin (an ER protein). Nuclei were stained with DAPI (blue). Scale bars, 10 µm.

# Supplementary Figure 5

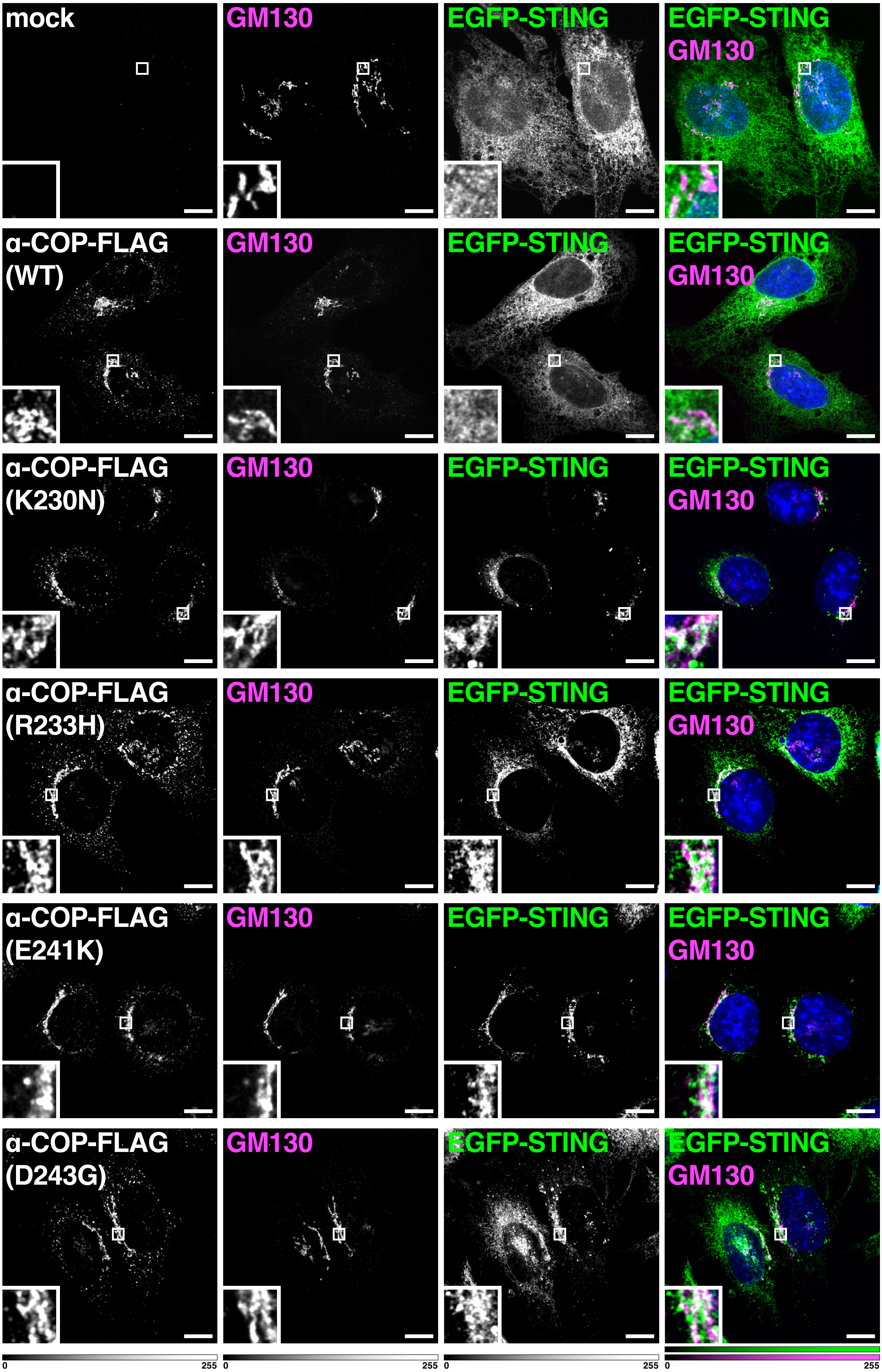


**Supplementary Figure 5 | STING translocates to the Golgi in the α-COP variants-expressing cells.**
α-COP-FLAG and EGFP-STING were stably expressed in *Sting*$^{-/-}$ MEFs. Cells were fixed, permeabilized, and stained for GM130 (a Golgi protein). Nuclei were stained with DAPI (blue). Scale bars, 10 µm.

# Supplementary Figure 6

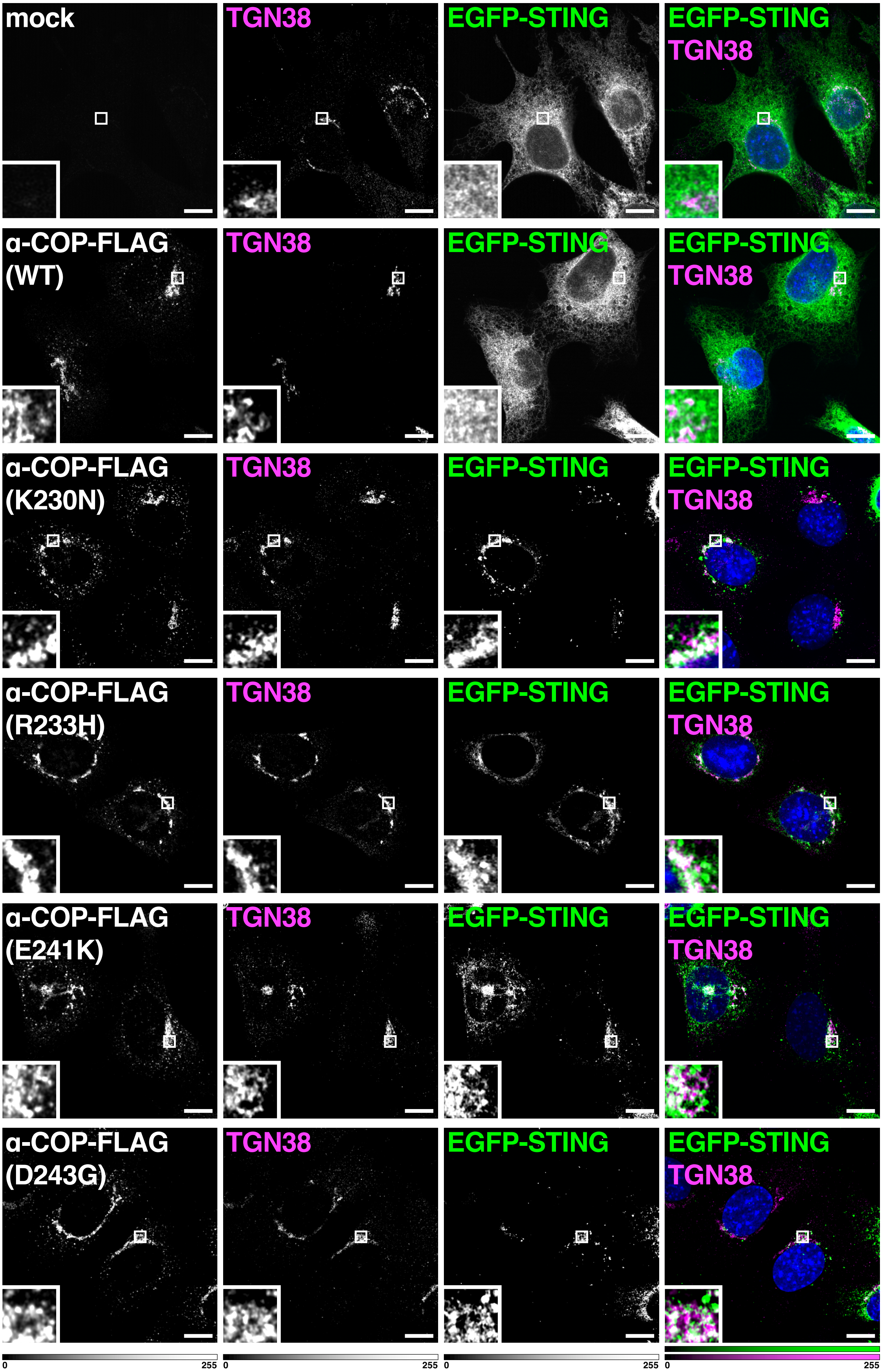


**Supplementary Figure 6 | STING translocates to the Golgi in the α-COP variants-expressing cells.**
High resolution images of Fig. 2a. Scale bars, 10 μm.

# Supplementary Figure 7

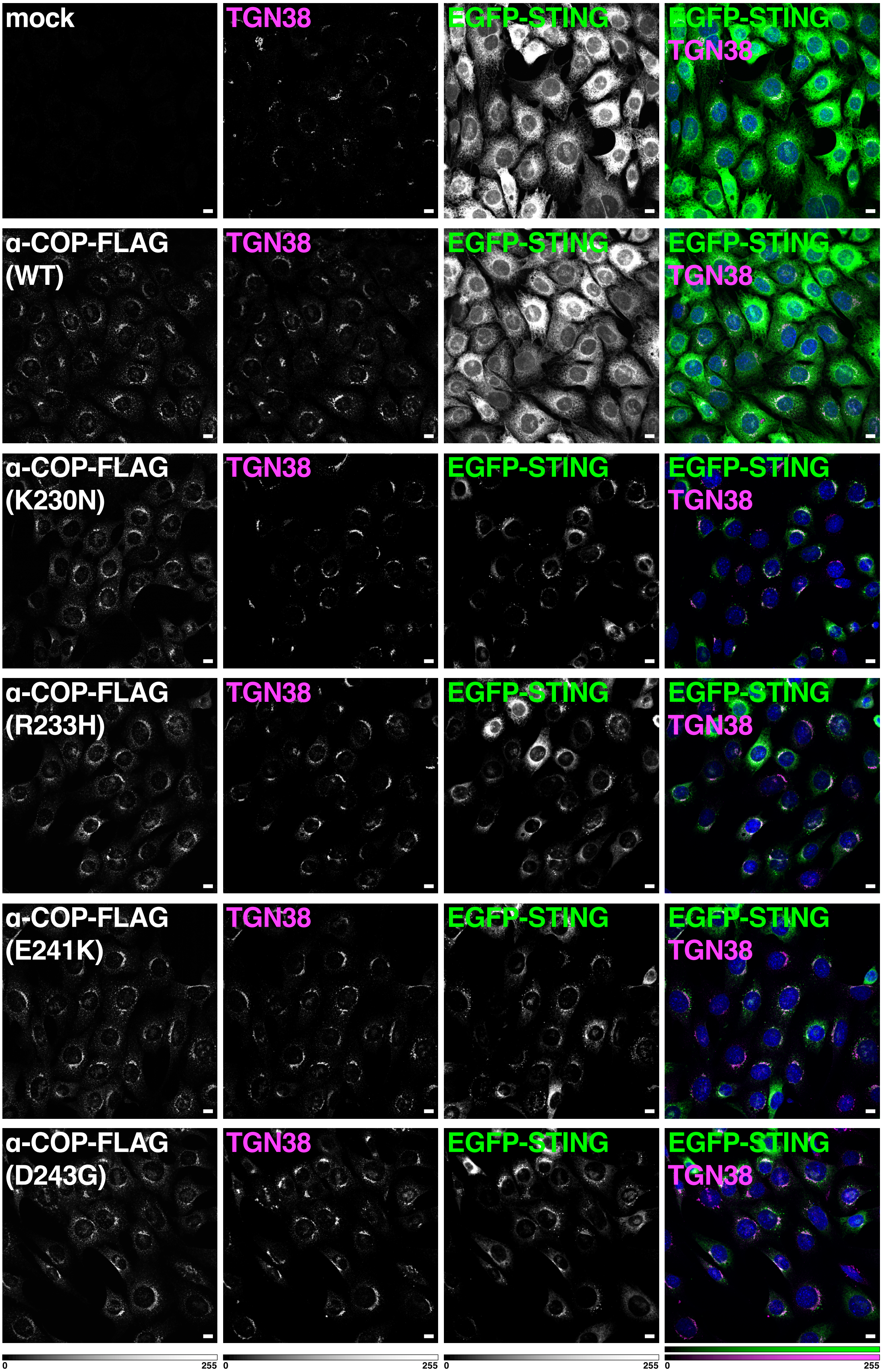


**Supplementary Figure 7 | Wider view images of the cells related to Fig.2a.**
α-COP-FLAG and EGFP-STING were stably expressed in *Sting*$^{-/-}$ MEFs. Cells were fixed, permeabilized, and stained for TGN38 (a Golgi protein). Nuclei were stained with DAPI (blue). Scale bars, 10 μm.

# Supplementary Figure 8

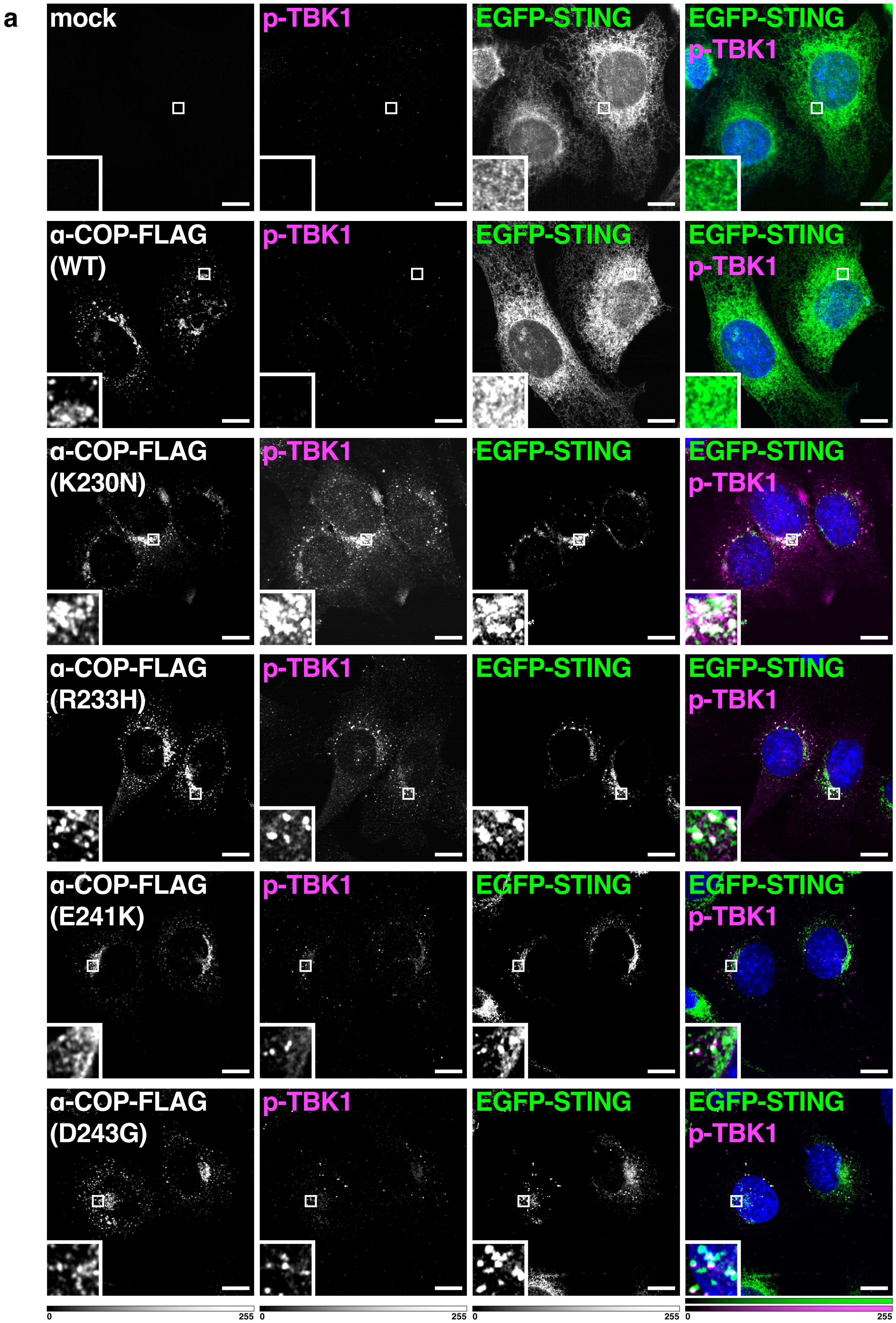

# Supplementary Figure 8 (continued)

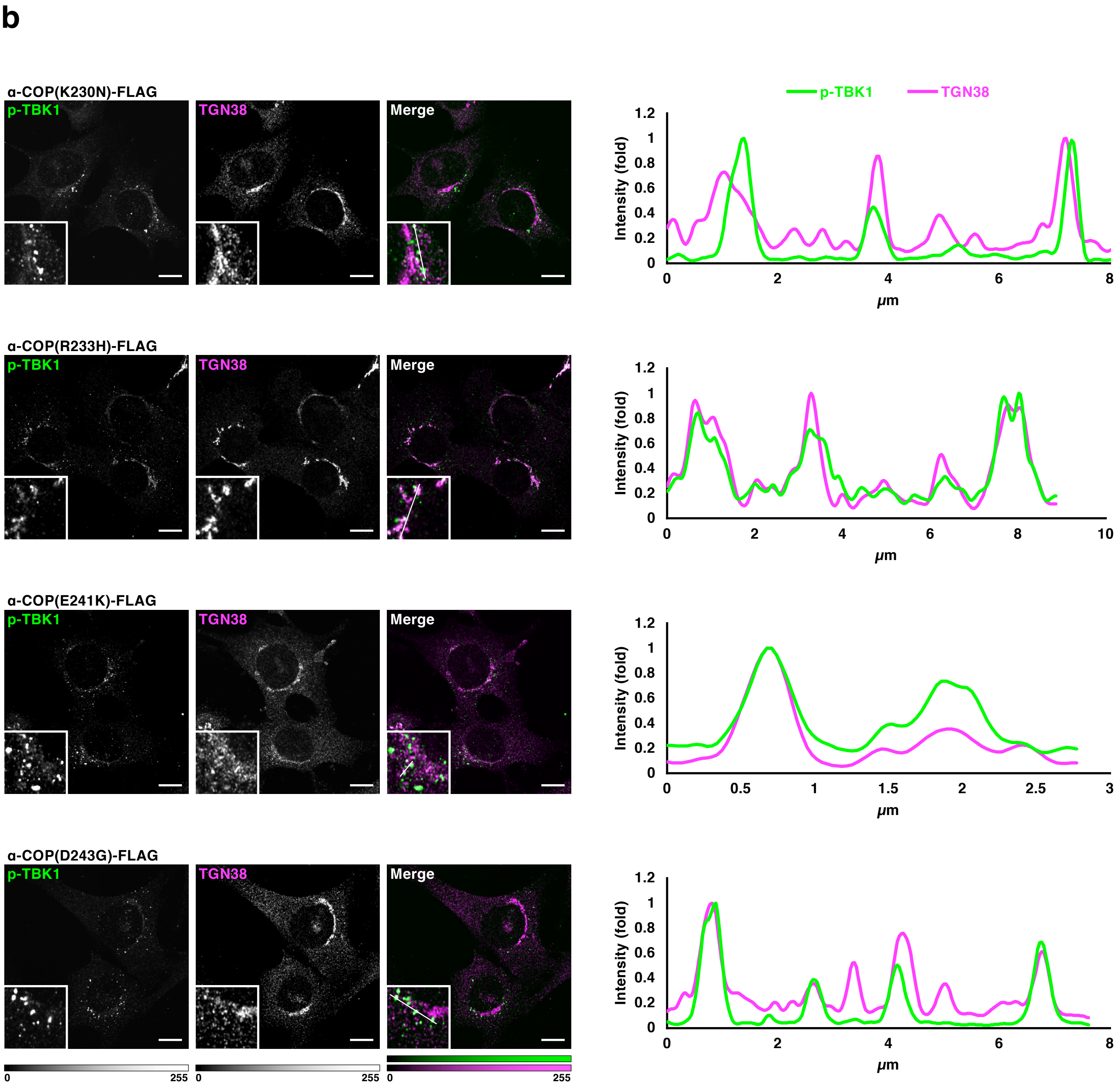


**Supplementary Figure 8 | STING activates TBK1 at TGN in the α-COP variants-expressing cells.**
**a**, High resolution images of Fig. 2b. Images of p-TBK1 were taken in the same conditions and displayed with the same contrast levels. **b**, The α-COP variants and EGFP-STING-expressing *Sting*$^{-/-}$ MEFs were fixed, permeabilized, and stained for TGN38 (a Golgi protein) and p-TBK1. The magnified image of the perinuclear region is shown in the lower left box. Scale bars, 10 μm. Fluorescence intensity profile along the white line is shown in the right panel.

# Supplementary Figure 9

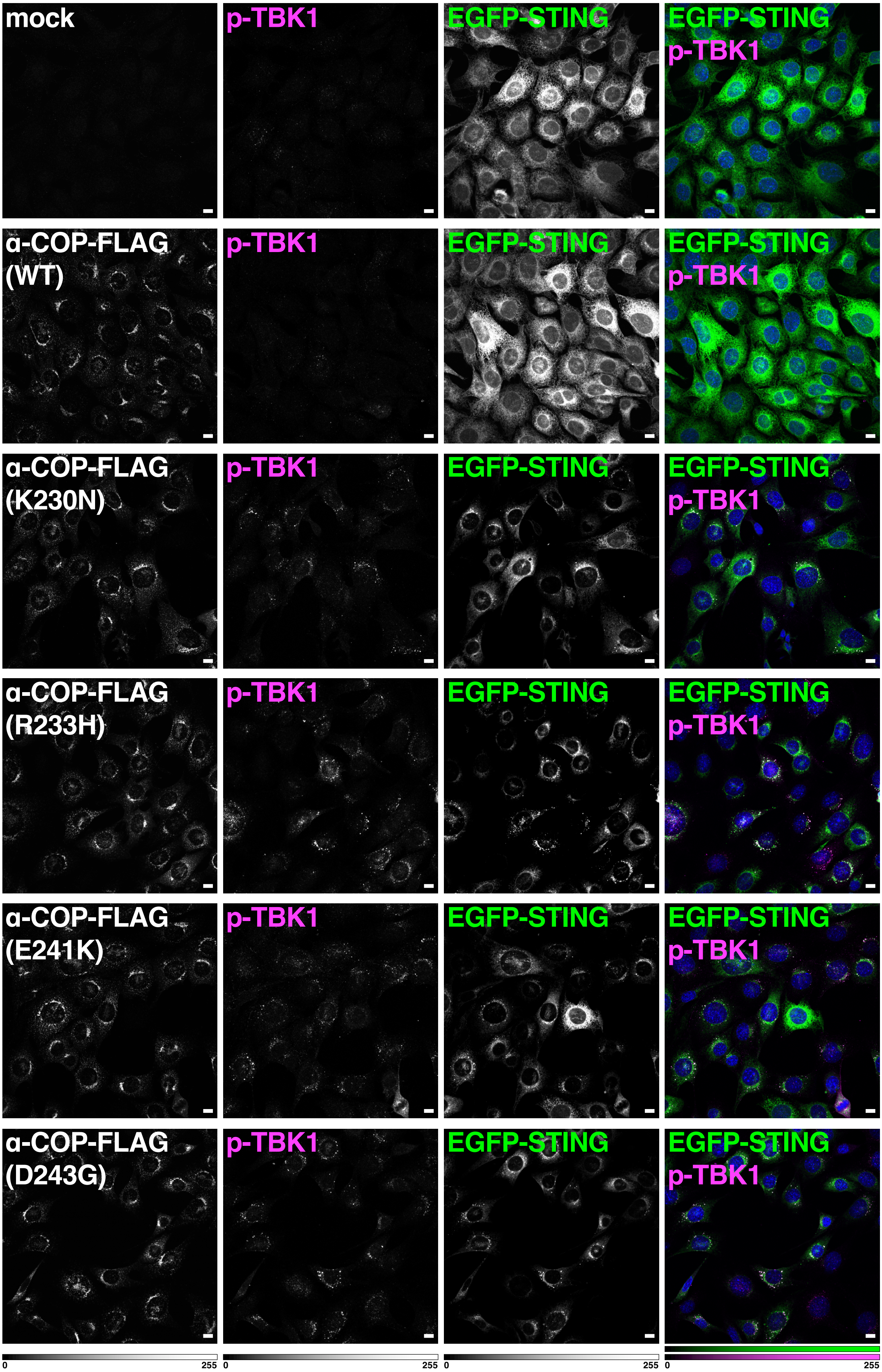


**Supplementary Figure 9 | Wider view images of the cells related to Fig.2b.**
α-COP-FLAG and EGFP-STING were stably expressed in *Sting*$^{-/-}$ MEFs. Cells were fixed, permeabilized, and stained for phospho-TBK1 (p-Ser172). Nuclei were stained with DAPI (blue). Scale bars, 10 µm. Images of p-TBK1 were taken in the same conditions and displayed with the same contrast levels.

# Supplementary Figure 10

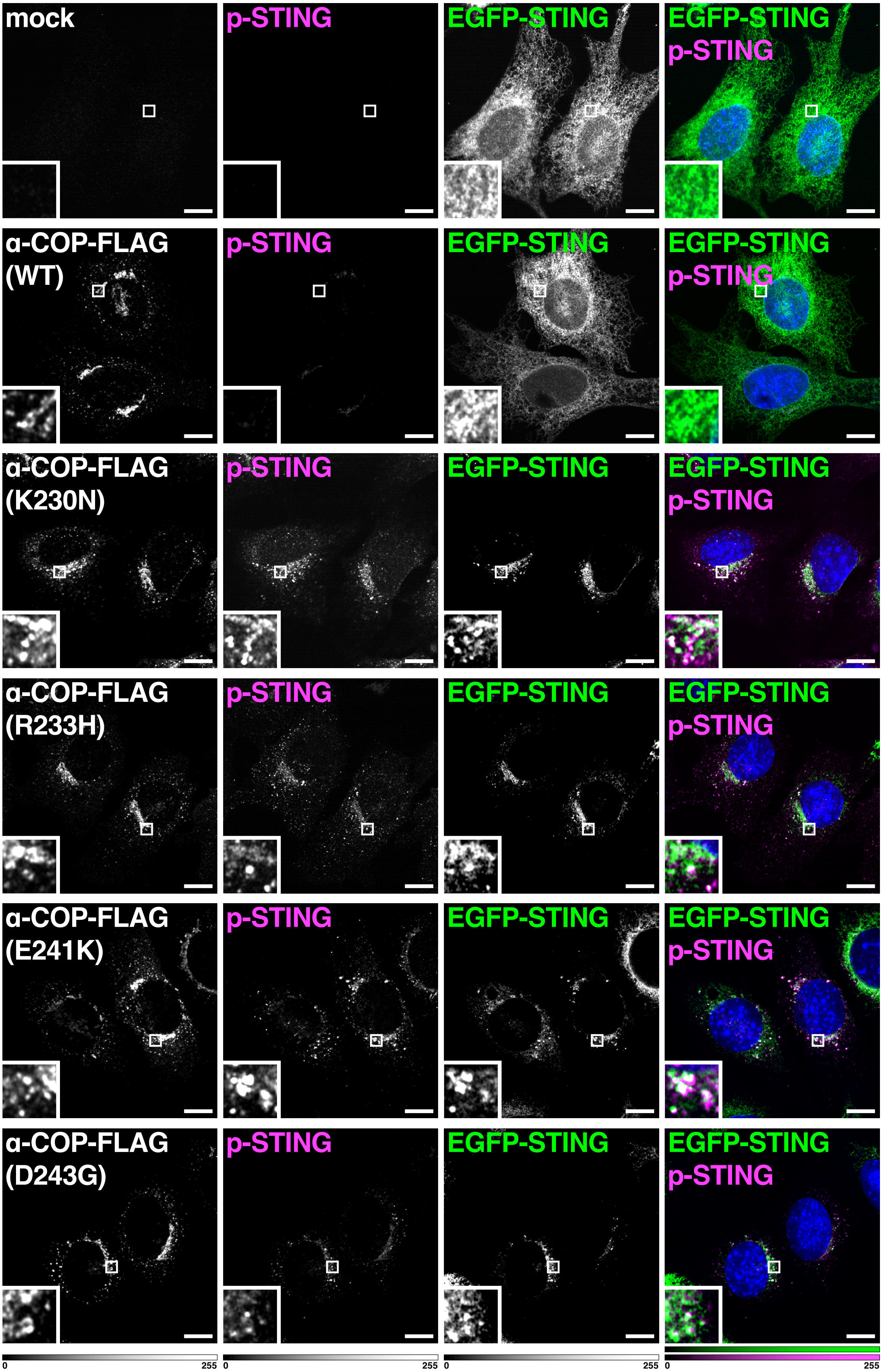


**Supplementary Figure 10 | Phosphorylated STING at Ser365 is detected at perinuclear compartments in the α-COP variants-expressing cells.** α-COP-FLAG and EGFP-STING were stably expressed in *Sting*$^{-/-}$ MEFs. Cells were fixed, permeabilized, and stained for phospho-STING (p-Ser365). Nuclei were stained with DAPI (blue). Scale bars, 10 μm. Images of p-STING were taken in the same conditions and displayed with the same contrast levels.

# Supplementary Figure 11

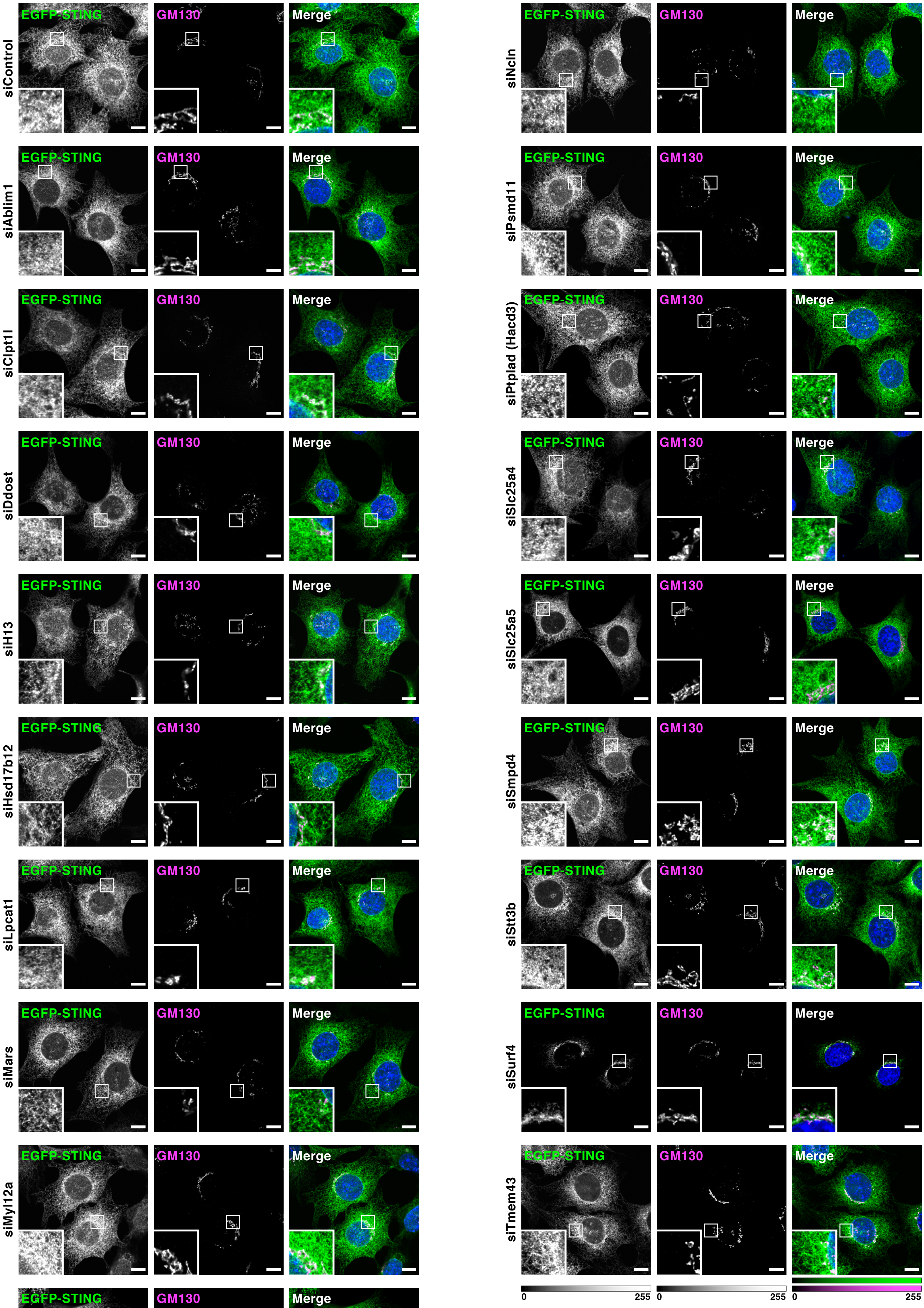


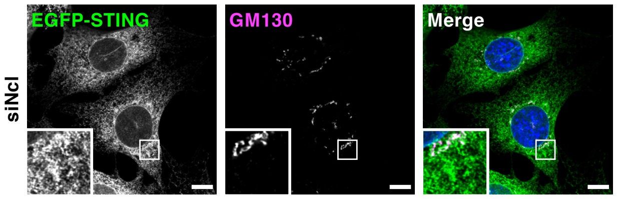


**Supplementary Figure 11 | Effects of siRNA knockdown of STING-binding proteins on subcellular localization of STING.**
EGFP-STING was stably expressed in *Sting*$^{-/-}$ MEFs. Cells were treated with siRNA (20 μM) as indicated for 48 h, fixed, permeabilized, and stained for GM130 (a Golgi protein). Nuclei were stained with DAPI (blue). Scale bars, 10 μm.

# Supplementary Figure 12

**a**

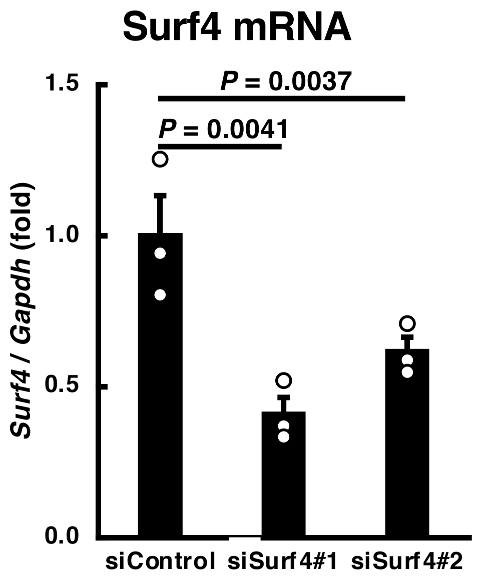


**b**

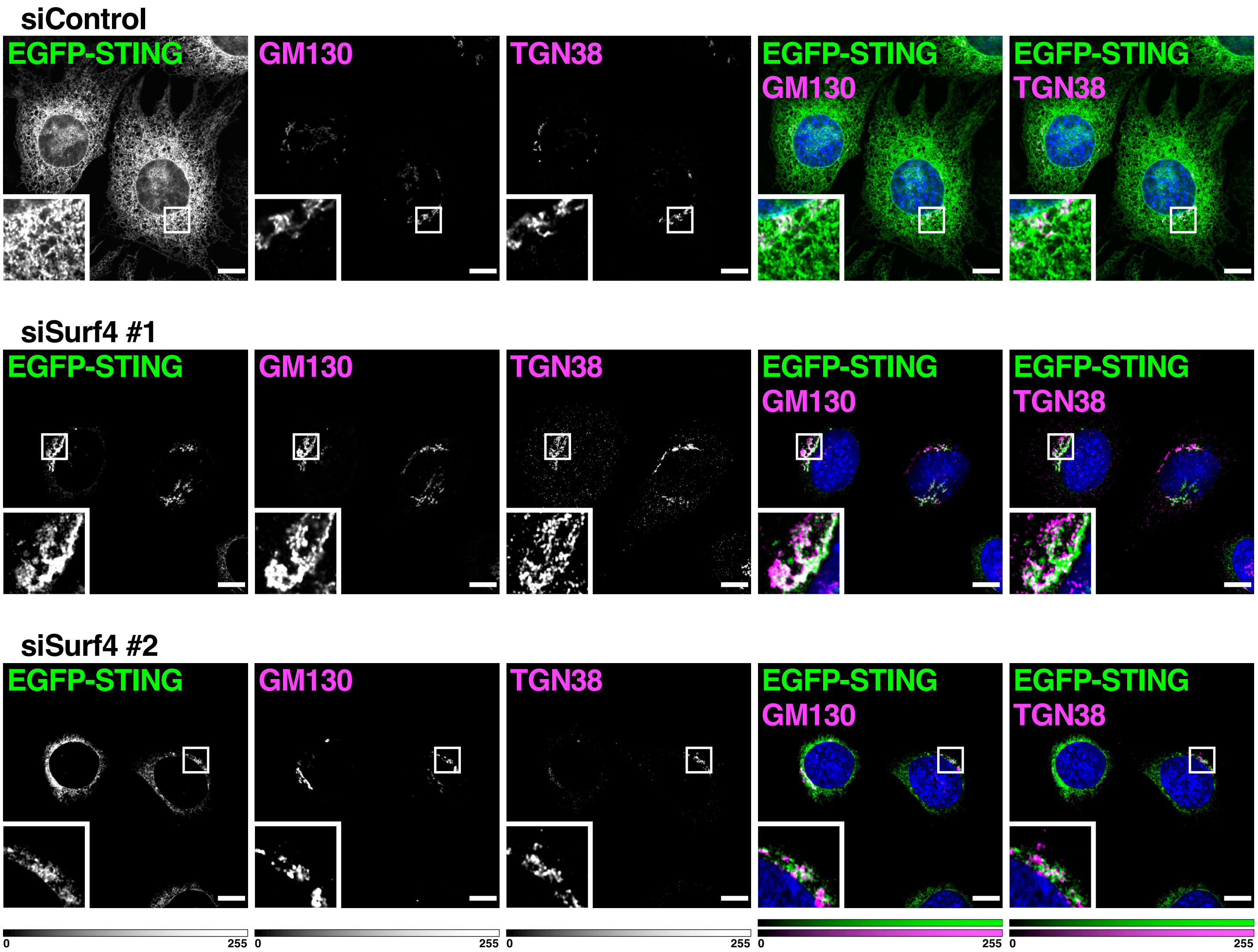


**Supplementary Figure 12 | Supplementary data related to Surf4 knockdown.**
**a**, EGFP-STING was stably expressed in *Sting*$^{-/-}$ MEFs. Cells were treated with siRNA (20 μM) as indicated for 48 h and qRT-PCR of the expression of Surf4 was performed. Data represent mean±s.e.m. of three independent experiments. Statistical significances were determined with one-way analysis of variance followed by Tukey–Kramer *post hoc* test. (one-way analysis of variance). **b**, High resolution images of Fig. 3c. Scale bars, 10 μm.

# Supplementary Figure 13

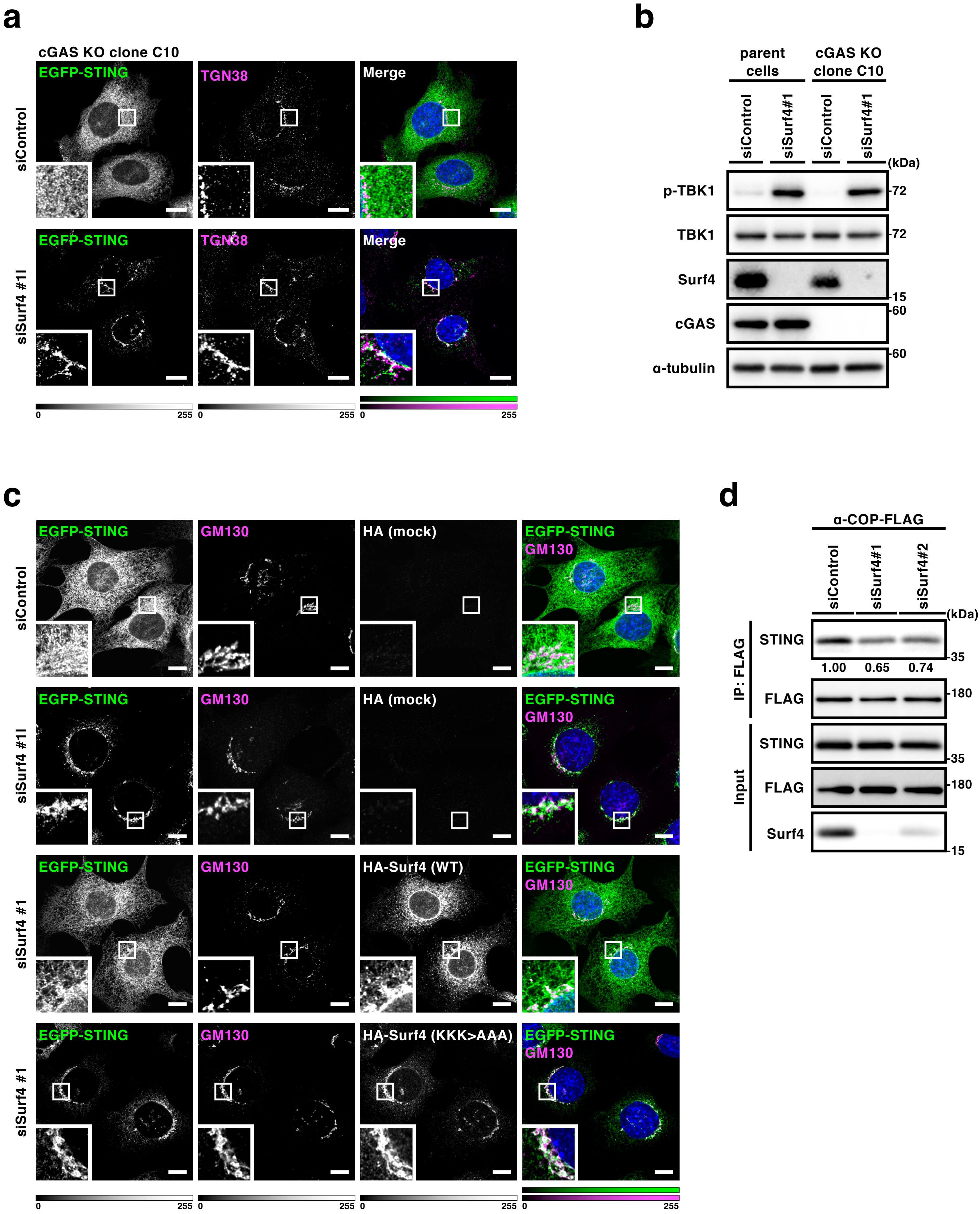


**Supplementary Figure 13 | Surf4 knockdown/rescue experiments.**

**a**, cGAS-knockout MEFs were generated by CRISPR-Cas9 system with *Sting*$^{-/-}$ MEFs, and EGFP-STING were then stably expressed in cGAS KO *Sting*$^{-/-}$ MEFs. Cells were treated with siRNA (20 μM) as indicated for 48 h. Cells were fixed, permeabilized, and stained for TGN38 (a Golgi protein). Nuclei were stained with DAPI (blue). Scale bars, 10 μm. **b**, siRNA-treated cells were prepared as in (**a**) and cell lysates were analyzed by western blot. **c**, EGFP-STING and siRNA-resistant HA-Surf4 (WT or K265A/K266A/K267A) were stably expressed in *Sting*$^{-/-}$ MEFs. Cells were treated with siRNA (20 μM) as indicated for 48 h, fixed, permeabilized, and stained for GM130 (a Golgi protein). Nuclei were stained with DAPI (blue). Scale bars, 10 μm. **d**, α-COP-FLAG was stably expressed in MEFs and the cells were treated with siRNA (20 μM) as indicated for 48 h. Cell lysates were prepared, and α-COP-FLAG was immunoprecipitated. Cell lysates and the immunoprecipitates were analyzed by western blot.

# Supplementary Figure 14

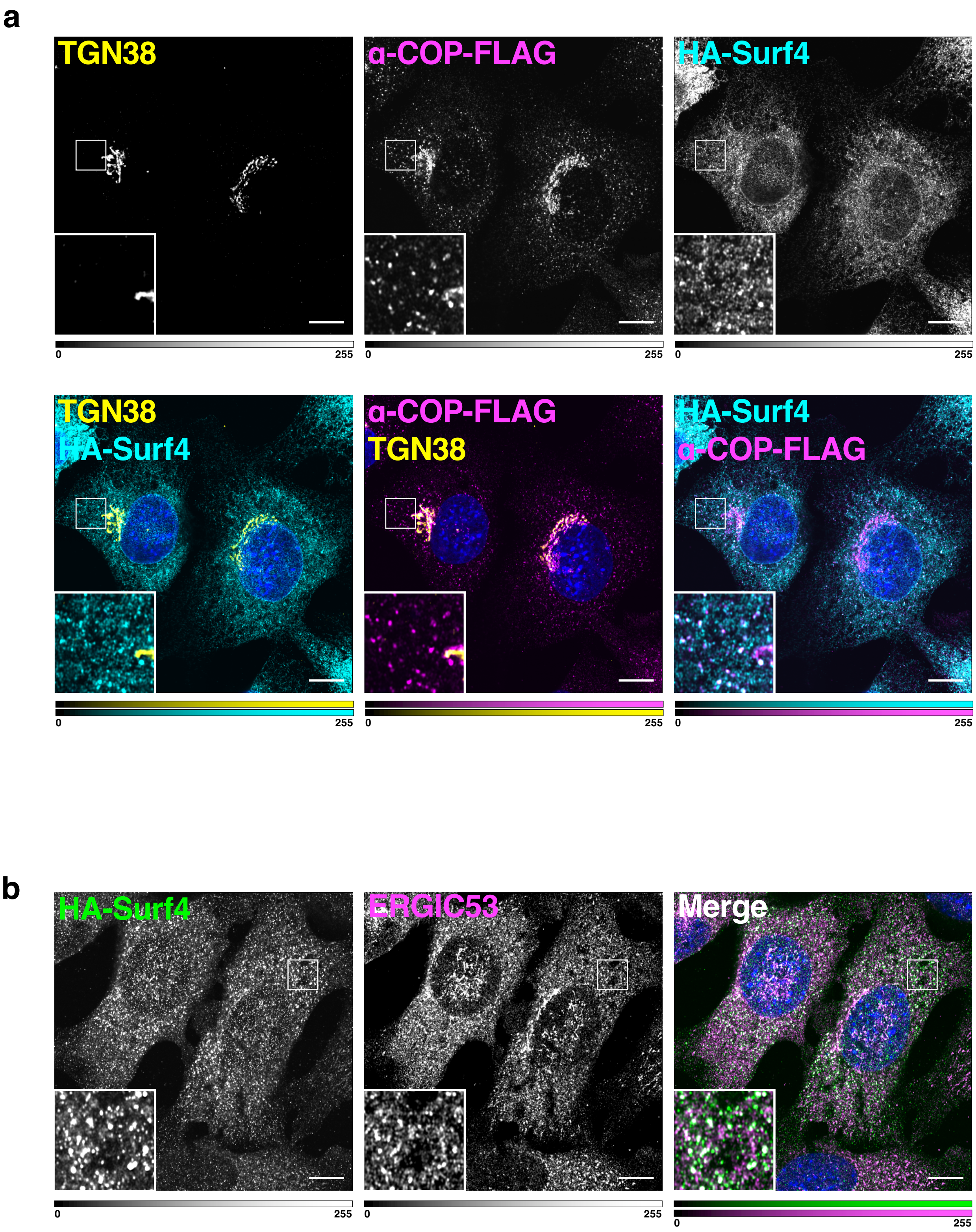


**Supplementary Figure 14 | Subcellular localization of Surf4 and α-COP.**
a, α-COP-FLAG and HA-Surf4 were stably expressed in MEFs. Cells were fixed, permeabilized, and stained for FLAG (α-COP-FLAG), HA (HA-Surf4), and TGN38 (a Golgi protein). Nuclei were stained with DAPI (blue). Scale bars, 10 µm. b, HA-Surf4 were stably expressed in MEFs. Cells were fixed, permeabilized, and stained for HA (HA-Surf4) and ERGIC53. Nuclei were stained with DAPI (blue). Scale bars, 10 µm.

# Supplementary Figure 15

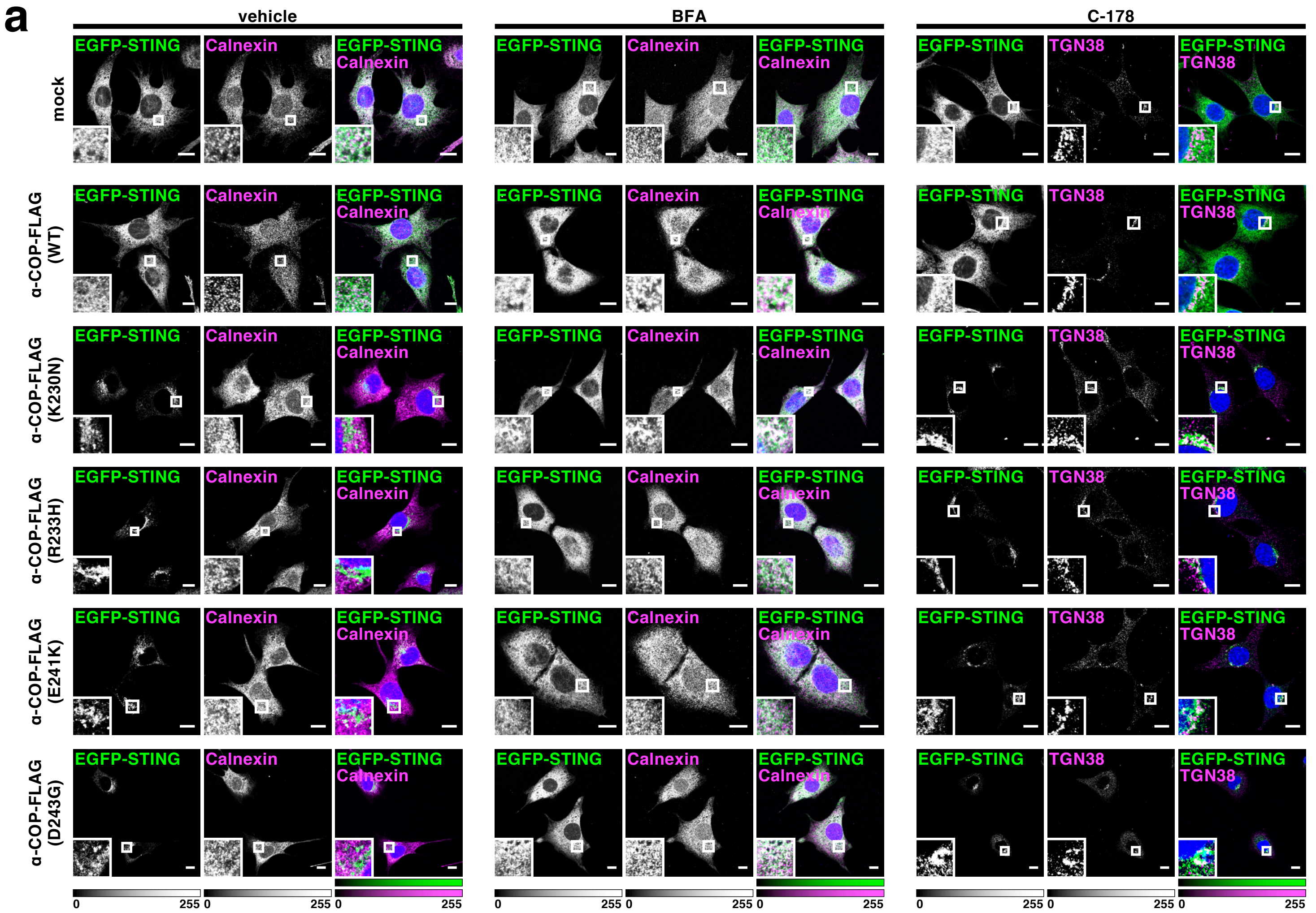


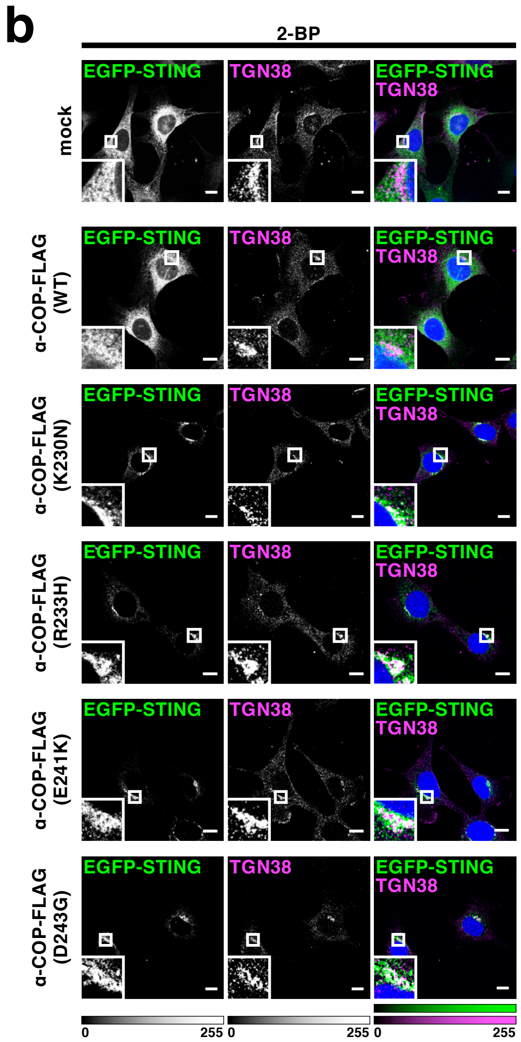


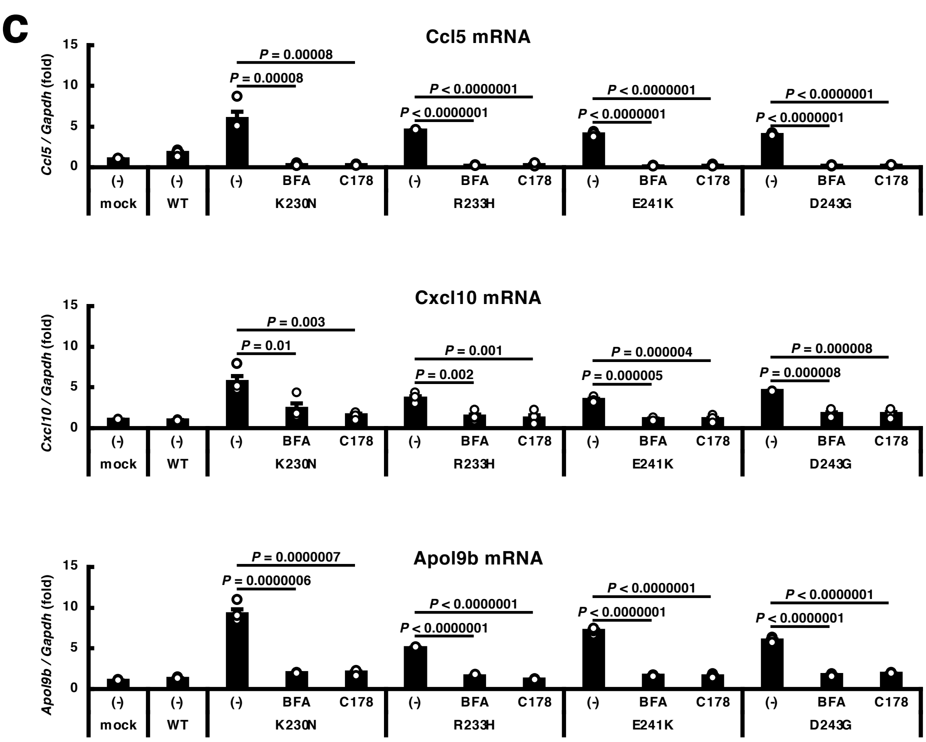


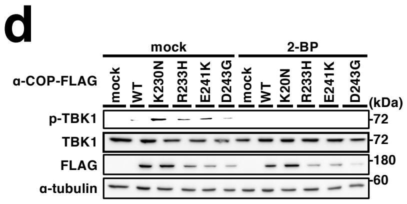


**Supplementary Figure 15 | The effect of BFA, C-178, or 2-BP treatment on the α-COP variants-expressing cells.**
**a**,**b**, α-COP-FLAG and EGFP-STING were stably expressed in *Sting*$^{-/-}$ MEFs. Cells were treated with BFA (0.3 μg/mL), C178 (10 μM) (**a**), or 2-BP (100 μM) (**b**) for 8 h, fixed, permeabilized, and stained for calnexin (an ER protein) or TGN38 (a Golgi protein). Nuclei were stained with DAPI (blue). Scale bars, 10 μm. **c**, qRT-PCR of the expression of indicated genes in α-COP-expressing MEFs that were treated with BFA (0.3 μg/mL) or C178 (10 μM) for 16 h. **d**, α-COP-FLAG and EGFP-STING were stably expressed in *Sting*$^{-/-}$ MEFs. Cells were treated with 2-BP (100 μM) for 8 h. Cell lysates were prepared and analyzed by western blot. Data in **c** are mean±s.e.m. from four independent experiments. Statistical significances were determined with one-way analysis of variance followed by Tukey–Kramer *post hoc* test.

# Supplementary Figure 16

**a**

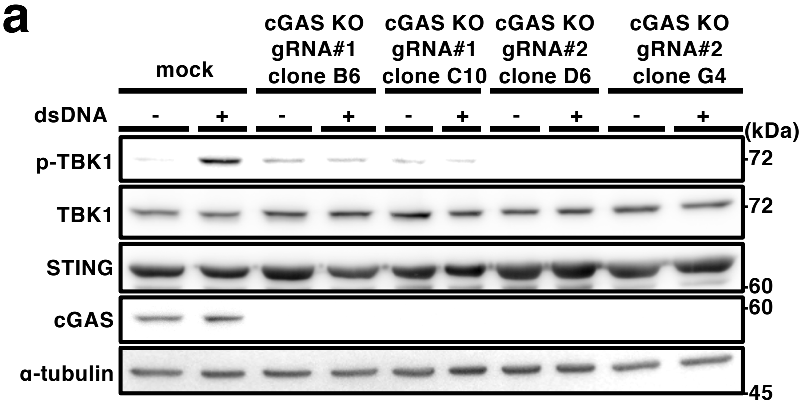


**d**

| | | *Ccl5* | *Cxcl10* | *Apol9b* |
|---|---|---|---|---|
| C10 | WT-K230N | 0.0000259 | 0.0000004 | 0.0000001 |
| | WT-R233H | 0.0000729 | 0.0004422 | 0.0000158 |
| | WT-E241K | < 0.0000001 | 0.0000038 | < 0.0000001 |
| | WT-D243G | < 0.0000001 | 0.0000039 | < 0.0000001 |
| D6 | WT-K230N | < 0.0000001 | < 0.0000001 | < 0.0000001 |
| | WT-R233H | 0.0000039 | 0.0000189 | 0.0000087 |
| | WT-E241K | < 0.0000001 | 0.0000097 | 0.0000012 |
| | WT-D243G | < 0.0000001 | 0.0000002 | < 0.0000001 |

**b**

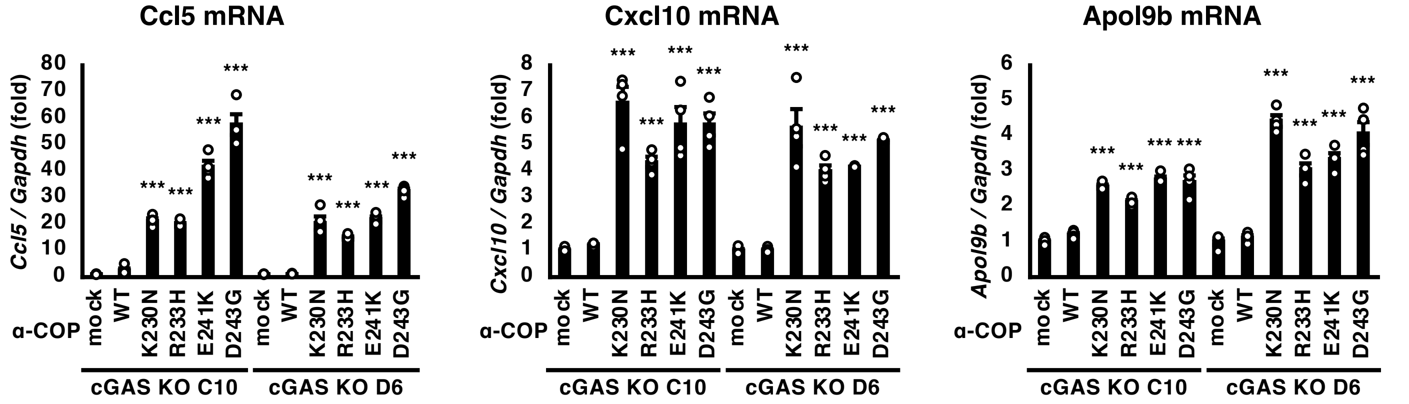


**c**

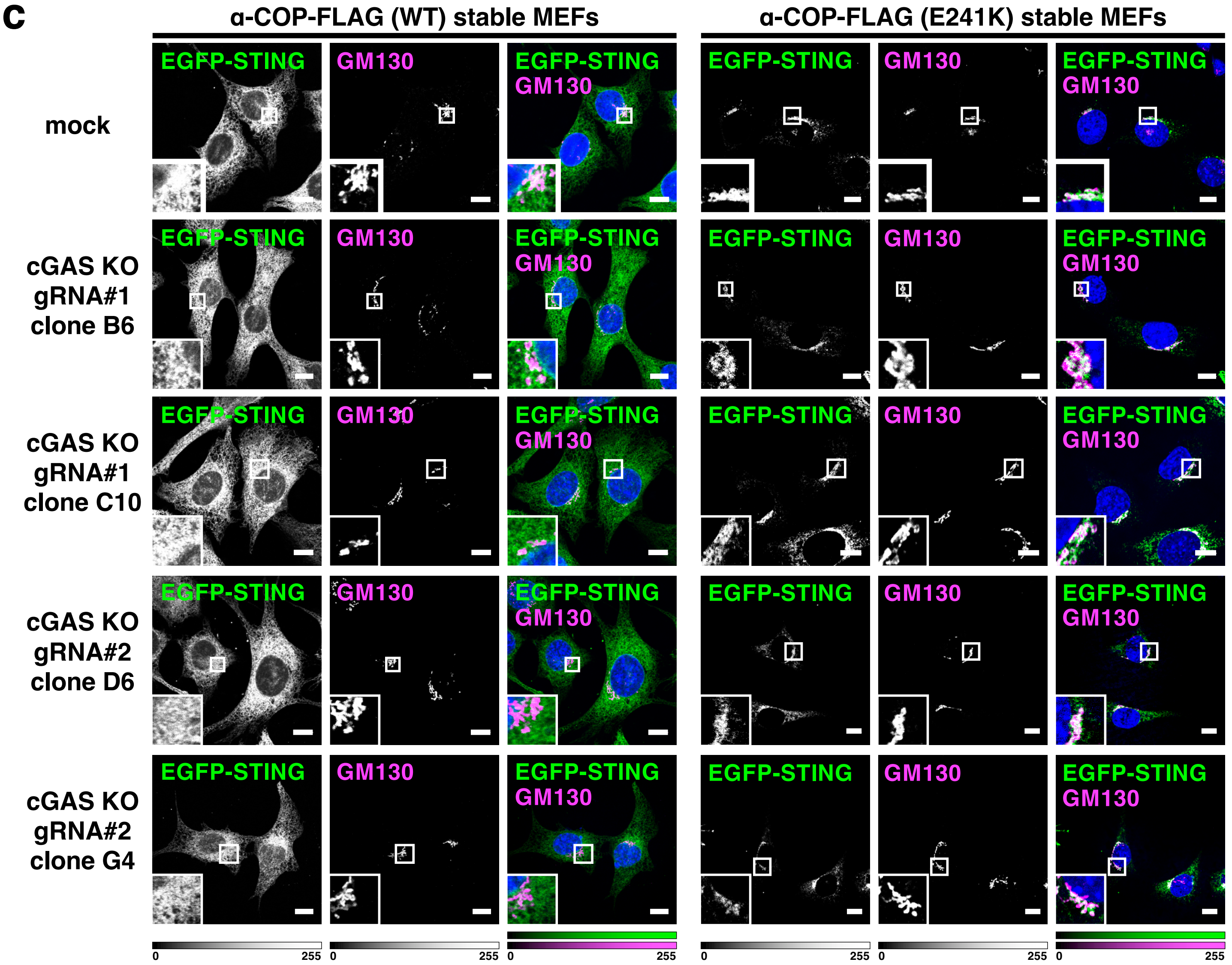


**Supplementary Figure 16 | STING translocates to the Golgi and innate immune genes are induced in the α-COP variants-expressing cGAS-KO cells.**
**a**, cGAS-knockout MEFs were generated by CRISPR-Cas9 system with *Sting*$^{-/-}$ MEFs. α-COP-FLAG and EGFP-STING were then stably expressed in cGAS-KO *Sting*$^{-/-}$ MEFs. Cells were stimulated with dsDNA for 2 h. Cell lysates were prepared and analyzed by western blot. **b**, qRT-PCR of the expression of indicated genes in the α-COP variant expressing cGAS-KO MEFs. Data are mean±s.e.m. from four independent experiments. Statistical significances between α-COP (WT) and α-COP variants were determined with one-way analysis of variance followed by Tukey–Kramer *post hoc* test. ***$P<0.001$. **c**, Cells were fixed, permeabilized, and stained for GM130 (a Golgi protein). Nuclei were stained with DAPI (blue). Scale bars, 10 μm. **d**, Exact *P* values in **b** are indicated.

# Supplementary Figure 17

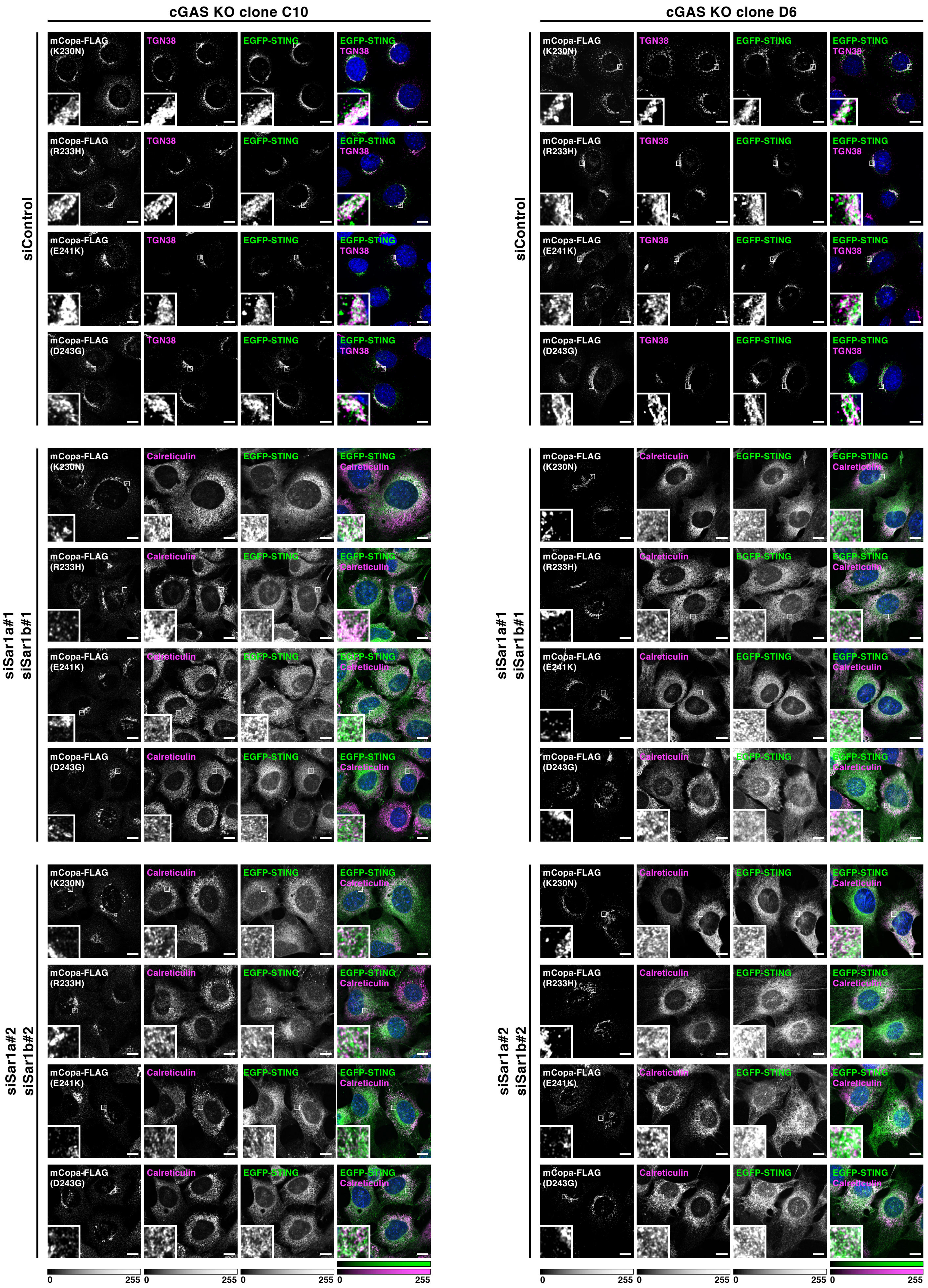


**Supplementary Figure 17 | Knockdown of Sar1 in cGAS KO cells inhibits the α-COP variants-induced translocation of STING to the Golgi.**

cGAS-knockout MEFs were generated by CRISPR-Cas9 system with *Sting*$^{-/-}$ MEFs, and EGFP-STING and α-COP variants were then stably expressed in cGAS-KO *Sting*$^{-/-}$ MEFs. Cells were treated with siRNA (20 μM) as indicated for 48 h. Cells were fixed, permeabilized, and stained for TGN38 (a Golgi protein) or calreticulin (an ER protein). Nuclei were stained with DAPI (blue). Scale bars, 10 μm.

# Supplementary Figure 18

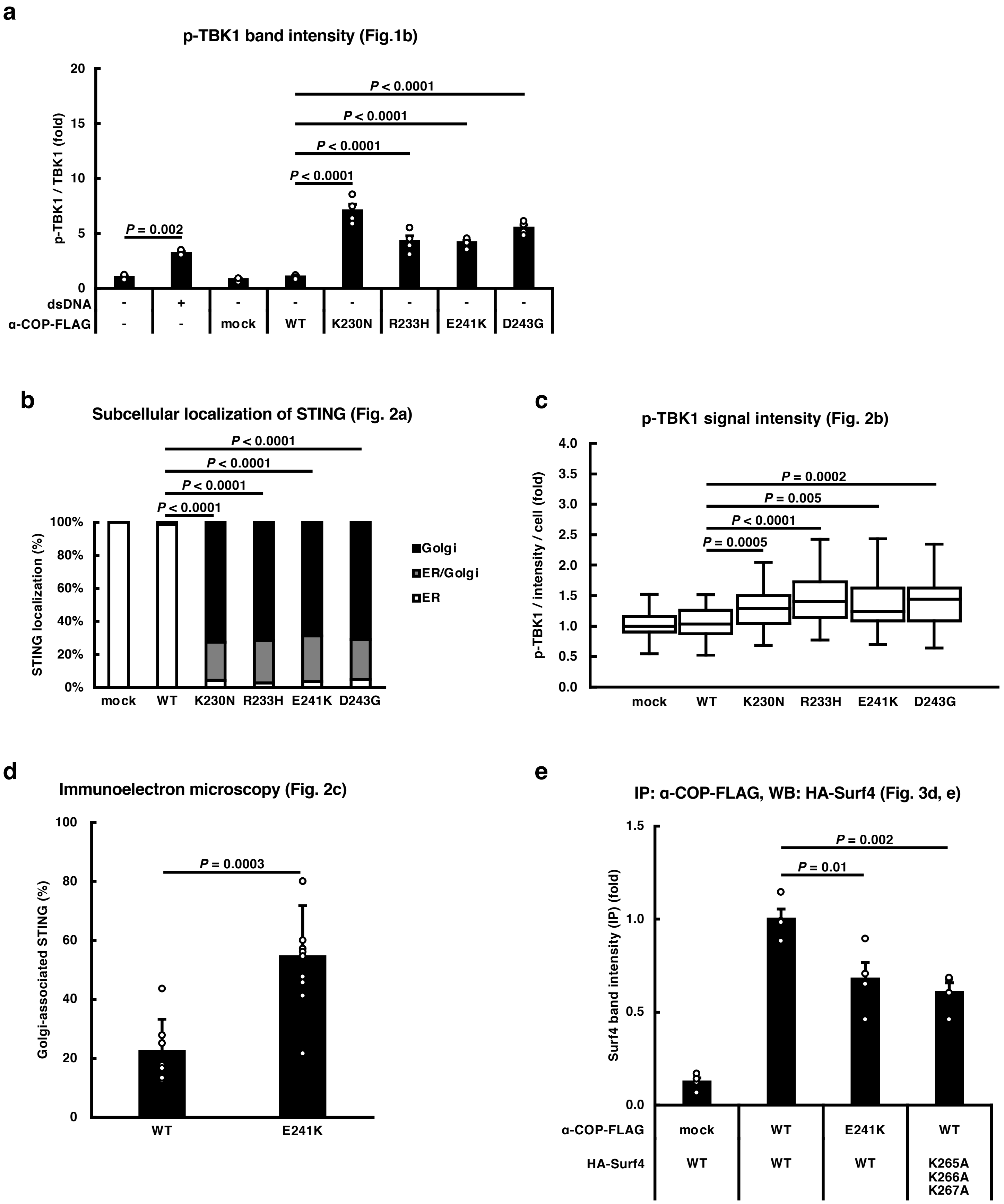


**Supplementary Figure 18 | Statistical analysis.**
**a**, The band intensities in Fig. 1b were quantified. The ratio of p-TBK1 to TBK1 was calculated and was normalized to the value from dsDNA(-)/mock experiment. Data represent mean±s.e.m. of five independent experiments. **b**, Subcellular localization of STING in cells in Fig. 2a was examined by a blinded observer (n > 100 cells from six independent experiments). "ER": STING mostly localized at the ER; "Golgi" STING mostly localized at perinuclear compartments that include the Golgi; "ER/Golgi": STING localized both the ER and perinuclear compartments (mock n = 185 cells; WT n = 164 cells; K230 N n = 117 cells; R233H n = 109 cells; E241K n = 119 cells; D243G n = 103 cells from six independent experiments). **c**, The fluorescence intensity of p-TBK1 in Fig. 2b was quantified and normalized to that of mock-transduced cells. Data are presented in box-and-whisker plot with the minimum, maximum, sample median, and first vs. third quartiles. (mock n = 60 cells; WT n = 46 cells; K230N n = 34 cells; R233H n = 41 cells; E241K n = 26 cells; D243G n = 33 cells from three independent experiments). **d**, In Fig. 2c, images of morphologically preserved region (1 ~ 4 μm$^2$) containing a profile of the Golgi stack with 6 nm colloidal gold particles indicating α-COP were taken. 12 nm colloidal gold particles indicating STING that were associated or not-associated with the Golgi stack were then counted. Percentage of the number was plotted as mean± s.d. for wild-type α-COP (n=7) and E241K α-COP (n=10). **e**, The band intensities of co-immunoprecipitated Surf4 in Fig. 3d, e were quantified and normalized to the value from mock/HA-Surf4 (WT) experiment. Data are mean±s.e.m. from four independent experiments. Statistical significances were determined with one-way analysis of variance followed by Tukey–Kramer *post hoc* test (**a**, **c**, **e**), Fisher's exact test with Holm's correction for multiple comparison (**b**), or two-tailed Student's *t*-test (**d**).

# Supplementary Figure 19

α-COP (WT)

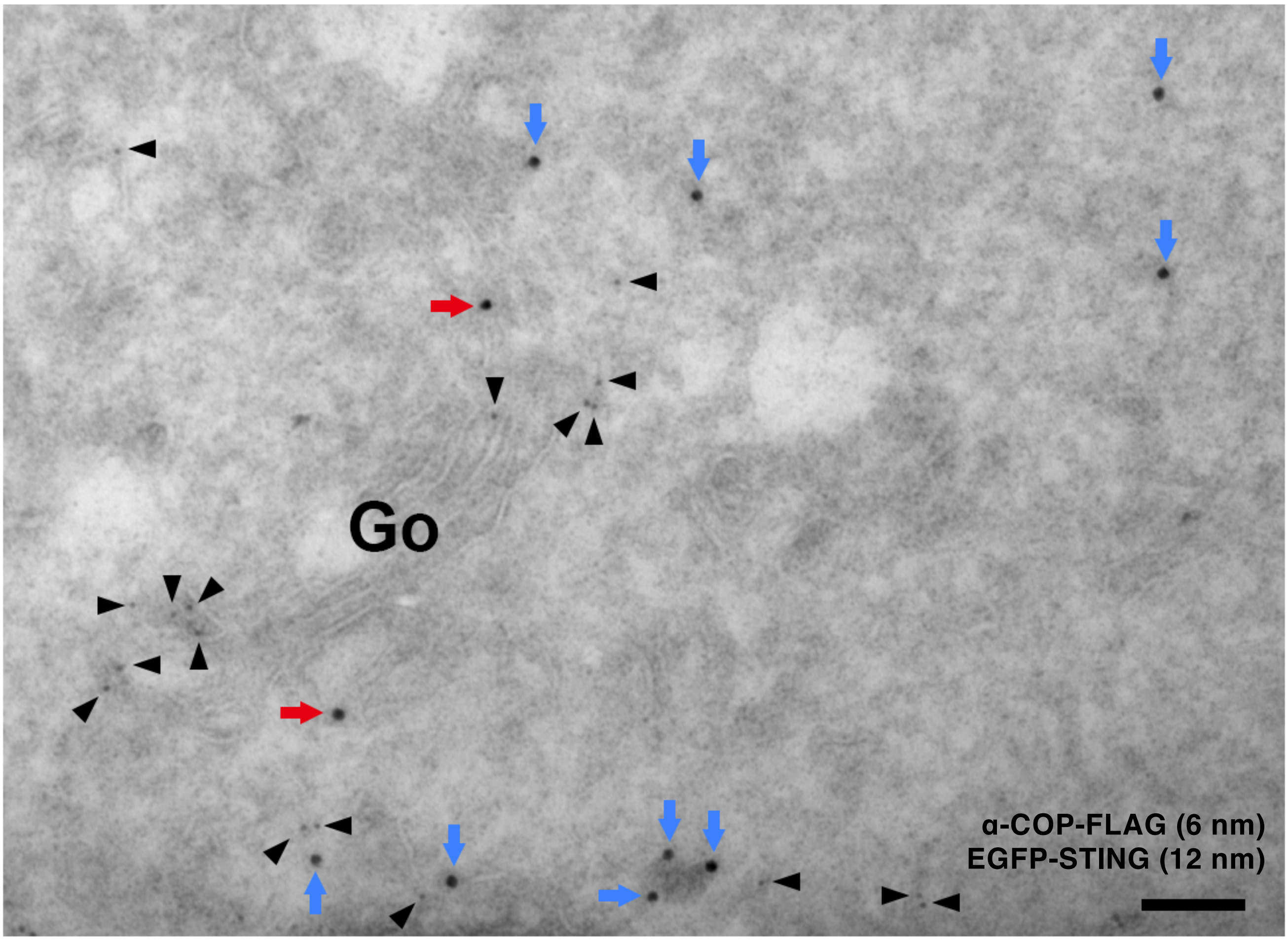


α-COP (E241K)

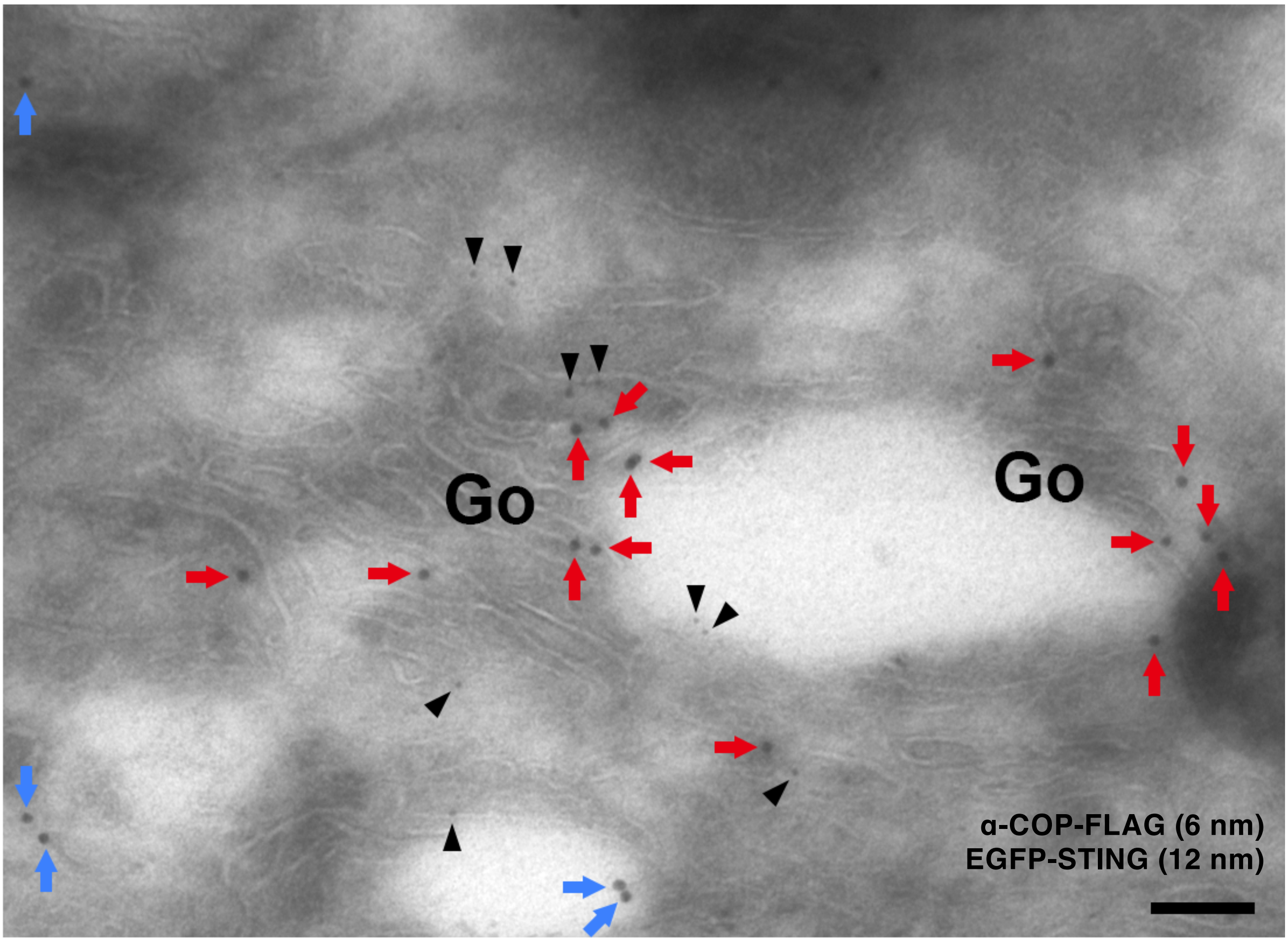


**Supplementary Figure 19 | High resolution images of Fig. 2c.**

Supplementary Table 1

| Gene Symbol | Entry | # Peptides | C terminus | KXKXX | KXHXX | KKXX |
|---|---|---|---|---|---|---|
| Ablim1 | Q8K4G5 | 1 | KAKLF | O | | |
| Clptm1l | Q8BXA5 | 1 | KPHPD | | O | |
| Ddost | O54734 | 7 | KEKSD | O | | |
| H13 | Q9D8V0 | 1 | EKKEK | | | O |
| Hsd17b12 | O70503 | 4 | KRKKN | O | | |
| Lpcat1 | Q3TFD2 | 2 | CKKAD | | | O |
| Mars | Q68FL6 | 1 | GKKKK | | | O |
| Myl12a | Q6ZWQ9 | 8 | KDKDD | O | | |
| Ncl | P09405 | 48 | KTKFE | O | | |
| Ncln | Q8VCM8 | 7 | KAKAQ | O | | |
| Psmd11 | Q8BG32 | 5 | AKKLT | | | O |
| Ptplad1; Hacd3 | Q8K2C9 | 3 | KKKLH | O | | O |
| Slc25a4 | P48962 | 20 | IKKYV | | | O |
| Slc25a5 | P51881 | 22 | IKKYT | | | O |
| Smpd4 | Q6ZPR5 | 3 | KLHQL | | O | |
| Stt3b | Q3TDQ1 | 1 | SKKTV | | | O |
| Surf4 | Q64310 | 4 | KKKEW | O | | O |
| Tmem43 | Q9DBS1 | 7 | AKKLE | | | O |

Cell lysates of FLAG-STING-reconstituted *Sting*$^{-/-}$ MEFs were prepared and FLAG-STING were immunoprecipitated with anti-FLAG M2 antibody. The immunoprecipitates were analyzed by mass spectrometry. Eighteen proteins with the COP-I binding motifs were identified and listed.

Supplementary Table 2

| | Forward primer (5’ > 3’) | Reverse primer (5’ > 3’) |
|---|---|---|
| *Ccl5* | ACCACTCCCTGCTGCTTTGCCT | GGCACACACTTGGCGGTTCCTT |
| *Ccxcl10* | AGTGCTGCCGTCATTTTCTGCCTC | GCAGGATAGGCTCGCAGGGATGATT |
| *Isg15* | GAGAGCAAGCAGCCAGAAGCAGAC | ACGGACACCAGGAAATCGTTACCCC |
| *Ifi44* | TCCCGAGCAGGATGAGGCAAACA | CGAGGAGGGCAGCTTCTGTCTCAGT |
| *Usp18* | GCAGAGAGCAGCAGGAGGAGCAAA | GGGCTGGACGAAACATCTCAAGGCA |
| *Ifih1* | GCAACATGGGCAGGGATTCAGGCA | GCAGTTCTGGCTCGGGGGATACTCT |
| *Neurl3* | AGCCAACGCCTGGATTCGTAGTGG | AGGCGGGTGTTGGCAGTGTTGT |
| *Stat2* | TGGCCCTACCCAGTTGGCTGAGATG | GCTGCTGGCTCTCCACAACTGCTTC |
| *Ddx58* | ACTGGGCGTGGCAGAACAAACC | ATCTCCGCTGGCTCTGAATGCCTC |
| *Zbp1* | TCACCCCAAGAAGGCAGGGCAA | GCTGAGGAGGACTGCCAAAGCAAAG |
| *Cxcl11* | ACCTGACCCTCTGCTGTCTTGGAAC | ACCACAGAAGGTAGCGTGGAGTGTG |
| *Xaf1* | GGCCTCTCTCCACTTCATGCTCCAC | GCAGGGTGCTGTTGGCTTTCCTT |
| *Gbp7* | TGATGAACCGCTTGGCAGGACAGA | TGTGAACTTGGGCTTGCTGGGGT |
| *Apol9b* | TTCGCTCCTCTGCCTCCTCATCCT | AGTGGCTCCCTGGATGGTGTGTCT |
| *Ifi27* | TGGGCTTCACTGGGACAGGCATT | AGGACCCCTGCTGATTGGAGTGTG |
| *Ifi44l* | TGAGGGCTGCGGATGATGCCTT | AATGGTCCCCAAACACCCCAGTGAA |
