# Supplementary material for: Homeostatic regulation of STING by retrograde membrane traffic to the ER
Source: Nat Commun. 2021 Jan 4;12:61. doi: 10.1038/s41467-020-20234-9 (PMC7782846; doi:10.1038/s41467-020-20234-9)
Supplement: Supplementary file 3 — Reporting Summary [file 41467_2020_20234_MOESM3_ESM.pdf]

nature research

Corresponding author(s): Tomohiko Taguchi

Last updated by author(s): Nov 10, 2020

Reporting Summary

Nature Research wishes to improve the reproducibility of the work that we publish. This form provides structure for consistency and transparency in reporting. For further information on Nature Research policies, see our Editorial Policies and the Editorial Policy Checklist.

Statistics

For all statistical analyses, confirm that the following items are present in the figure legend, table legend, main text, or Methods section.

n/a	Confirmed	
☐	☒	The exact sample size (*n*) for each experimental group/condition, given as a discrete number and unit of measurement
☐	☒	A statement on whether measurements were taken from distinct samples or whether the same sample was measured repeatedly
☐	☒	The statistical test(s) used AND whether they are one- or two-sided<br>*Only common tests should be described solely by name; describe more complex techniques in the Methods section.*
☐	☒	A description of all covariates tested
☐	☒	A description of any assumptions or corrections, such as tests of normality and adjustment for multiple comparisons
☐	☒	A full description of the statistical parameters including central tendency (e.g. means) or other basic estimates (e.g. regression coefficient) AND variation (e.g. standard deviation) or associated estimates of uncertainty (e.g. confidence intervals)
☐	☒	For null hypothesis testing, the test statistic (e.g. F, t, r) with confidence intervals, effect sizes, degrees of freedom and P value noted<br>*Give P values as exact values whenever suitable.*
☒	☐	For Bayesian analysis, information on the choice of priors and Markov chain Monte Carlo settings
☒	☐	For hierarchical and complex designs, identification of the appropriate level for tests and full reporting of outcomes
☒	☐	Estimates of effect sizes (e.g. Cohen's *d*, Pearson's *r*), indicating how they were calculated

Our web collection on statistics for biologists contains articles on many of the points above.

Software and code

Policy information about availability of computer code

Data collection	Zeiss ZEN 2.3 SP1 FP3 (black, 64 bit) (ver. 14.0.21.201)
Data analysis	Fiji (ver. 2.1.0/1.53c), R (ver. 4.0.2), MASCOT (ver. 2.6), Proteome Discoverer (ver. 2.2)

For manuscripts utilizing custom algorithms or software that are central to the research but not yet described in published literature, software must be made available to editors and reviewers. We strongly encourage code deposition in a community repository (e.g. GitHub). See the Nature Research guidelines for submitting code & software for further information.

Data

Policy information about availability of data

All manuscripts must include a data availability statement. This statement should provide the following information, where applicable:

- Accession codes, unique identifiers, or web links for publicly available datasets
- A list of figures that have associated raw data
- A description of any restrictions on data availability

The authors declare that the data supporting the findings of this study are available within the paper, Supplementary Information, and Source Data File or from the corresponding author upon request. The source data underlying Figs. 1, 2d, 3c-e, 4a-c, e-g, as well as Supplementary Figs. 1b-e, 2f, g, 3, 8b, 12a, 13b, d, 15c, d, 16a, b, and 18 are provided as a Source Data file.

Field-specific reporting

Please select the one below that is the best fit for your research. If you are not sure, read the appropriate sections before making your selection.

☒ Life sciences ☐ Behavioural & social sciences ☐ Ecological, evolutionary & environmental sciences

For a reference copy of the document with all sections, see nature.com/documents/nr-reporting-summary-flat.pdf

Life sciences study design

All studies must disclose on these points even when the disclosure is negative.

Sample size	No sample size calculation was applied in this study to predetermine sample sizes for experiments using cell lines. A sample size of three or more was used as to evaluate the spread of the data and was determined based upon other studies with similar methodologies (PMID: 27324217, 29093443).
Data exclusions	No data have been excluded from any analysis.
Replication	All experiments have been repeated at least three times independently, and each yielding similar results.
Randomization	No randomization was required for the presented experiments, because this study did not involve animals or human participants. All the experiments were performed with immortalized cell lines which can reasonably be assumed to be identical when split into multiple wells.
Blinding	The investigators were blinded during data analysis in Supplementary Figure 18b. Other experiments were unblinded because these experiments were not susceptible to bias.

Reporting for specific materials, systems and methods

We require information from authors about some types of materials, experimental systems and methods used in many studies. Here, indicate whether each material, system or method listed is relevant to your study. If you are not sure if a list item applies to your research, read the appropriate section before selecting a response.

Materials & experimental systems

n/a	Involved in the study	
☐	☒	Antibodies
☐	☒	Eukaryotic cell lines
☒	☐	Palaeontology and archaeology
☒	☐	Animals and other organisms
☒	☐	Human research participants
☒	☐	Clinical data
☒	☐	Dual use research of concern

Methods

n/a	Involved in the study	
☒	☐	ChIP-seq
☒	☐	Flow cytometry
☒	☐	MRI-based neuroimaging

Antibodies

Antibodies used	Antibodies used in this study were as follows: mouse anti-GFP (JL-8, dilution 1:1000) (Clontech); rabbit anti-β-COP (PA1-061, dilution 1:2000 for western blot and immunofluorescence), rabbit anti-mCherry for detecting mScarlet-I (PA5-34974, dilution 1:1000 for western blot), and Alexa 488-, 594-, or 647-conjugated secondary antibodies (A21202, A21203, A21206, A21207, A31573, A11016, A21448, dilution 1:2000) (Thermo Fisher Scientific); rabbit anti-TBK1 (ab40676, dilution 1:1000) (Abcam); rabbit anti-phospho-TBK1 (D52C2, dilution 1:1000 for western blot, dilution 1:100 for immunofluorescence), rabbit anti-cGAS (D3O8O, dilution 1:1000), and rabbit anti-phospho-STING (D1C4T, dilution 1:400) (Cell signaling); mouse anti-calreticulin (612136, dilution 1:1000), and mouse anti-GM130 (610823, dilution 1:1000) (BD Biosciences); rabbit anti-α-COP (HPA028024, dilution 1:1000 for western blot), mouse anti-α-tubulin (DM1A, dilution 1:5000) and mouse anti-FLAG M2 antibody (Sigma); Goat Anti-Rabbit IgG(H+L) Mouse/Human ads-HRP (4050-05, dilution 1:10000) and Goat Anti-Mouse IgG(H+L) Human ads-HRP (1031-05, dilution 1:10000) (Southern Biotech); sheep anti-TGN38 (AHR499G, dilution 1:500) (Serotec); rabbit anti-STING antibody (19851-1-AP, dilution 1:1000 for western blot), rabbit anti-γ-COP (12393-I-AP, dilution 1:1000 for western blot, dilution 1:200 for immunofluorescence), and rabbit anti-ERGIC53 (13364-1-AP, dilution 1:200 for immunofluorescence) (Proteintech); mouse anti-HA (4B2, dilution 1:1000 for western blot and immunofluorescence) and mouse anti-FLAG (1E6, dilution 1:1000 for western blot and immunofluorescence, dilution 1:50 for immunoelectron microscopy) (Wako); rabbit anti-β'-COP (A304-523A-T, dilution 1:1000 for western blot, dilution 1:200 for immunofluorescence) (Bethyl Laboratories); mouse anti-δ-COP (GTX630562, dilution 1:1000 for western blot, dilution 1:200 for immunofluorescence) (GeneTex); 12 nm colloidal gold particle-conjugated donkey anti-rabbit antibody (711-205-152, dilution 1:20) and 6 nm colloidal gold particle-conjugated donkey anti-mouse IgG (715-195-150, dilution 1:20) (Jackson ImmunoResearch laboratories). For the immunoprecipitation of FLAG-tagged protein, anti-FLAG M2 Affinity Gel (A2220, Sigma) was used. For the immunoprecipitation of GFP-tagged protein, anti-GFP nanobody was used. pGEX6P1-GFP-Nanobody was a gift from Kazuhisa Nakayama (Addgene plasmid # 61838).

Validation	All antibodies were validated by the vendors and documented with corresponding data sheets as follows.<br>mouse anti-GFP (JL-8, Clontech): Validated for WB with cell lysate.<br>rabbit anti-β-COP (PA1-061, Thermo Fisher Scientific): Validated for mouse β-COP by WB with cell lysate.<br>rabbit anti-mCherry (PA5-34974, Thermo Fisher Scientific) : Validated for WB with cell lysate.<br>rabbit anti-TBK1 (ab40676, Abcam): Validated for mouse TBK1 for WB with cell lysate.<br>rabbit anti-phospho-TBK1 (D52C2, Cell signaling): Validated for mouse p-TBK1 by WB with cell lysate and by IF with fixed cells.<br>rabbit anti-cGAS (D3O8O, Cell signaling): Validated for mouse cGAS by WB with cell lysate.<br>rabbit anti-phospho-STING (D1C4T, Cell signaling) : Validated for mouse p-STING by IF with fixed cells.<br>mouse anti-calreticulin (612136, BD Biosciences): Validated for mouse calreticulin by WB with cell lysate.<br>mouse anti-GM130 (610823, BD Biosciences): Validated for mouse GM130 by IF with fixed cells.<br>rabbit anti-α-COP (HPA028024, Sigma): Validated for mouse α-COP by WB with cell lysate.<br>mouse anti-α-tubulin (DM1A, Sigma): Validated for mouse α-tubulin by WB with cell lysate.<br>mouse anti-FLAG M2 antibody (Sigma): Validated for WB with cell lysate.<br>sheep anti-TGN38 (AHR499G, Serotec): Validated for mouse TGN38 by IF with fixed cells.<br>rabbit anti-STING antibody (19851-1-AP, Proteintech): Validated for mouse STING by WB with cell lysate.<br>rabbit anti-γ-COP (12393-I-AP, Proteintech): Validated for mouse γ-COP by WB with cell lysate and by IF with fixed cells.<br>rabbit anti-ERGIC53 (13364-1-AP, Proteintech): Validated for mouse ERGIC53 by IF with fixed cells.<br>mouse anti-HA (4B2, Wako): Validated for WB with cell lysate and for IF with fixed cells.<br>mouse anti-FLAG (1E6, Wako): Validated for WB with cell lysate and for IF with fixed cells.<br>rabbit anti-β'-COP (A304-523A-T, Bethyl Laboratories): Validated for mouse β'-COP by WB with cell lysate and by IF with fixed cells.<br>mouse anti-δ-COP (GTX630562, GeneTex): Validated for mouse δ-COP by WB with cell lysate and by IF with fixed cells.

Eukaryotic cell lines

Policy information about cell lines

Cell line source(s)	HEK293T cells were from ATCC. Immortalized MEFs were generated from C57B6/J mice.
Authentication	Authentication of HEK293T cells was performed by ATCC with the short tandem repeat profiling.
Mycoplasma contamination	Confirm that all cell lines were tested negative for mycoplasma contaminations.
Commonly misidentified lines (See ICLAC register)	No commonly misidentified cell lines were used in the study.
